# Systematic review of the values and preferences regarding the use of injectable pre‐exposure prophylaxis to prevent HIV acquisition

**DOI:** 10.1002/jia2.26107

**Published:** 2023-07-13

**Authors:** Lara Lorenzetti, Nhi Dinh, Ariane van der Straten, Virginia Fonner, Kathleen Ridgeway, Michelle Rodolph, Robin Schaefer, Heather‐Marie A. Schmidt, Rachel Baggaley

**Affiliations:** ^1^ Global Health and Population Research FHI 360 Durham North Carolina USA; ^2^ ASTRA Consulting Kensington California USA; ^3^ Center for AIDS Prevention Studies Department of Medicine University of California San Francisco California USA; ^4^ World Health Organization Global HIV Hepatitis and STI Programmes Geneva Switzerland; ^5^ UNAIDS Regional Office for Asia and the Pacific Bangkok Thailand

**Keywords:** acceptability, injectable PrEP, long‐acting injectable cabotegravir, pre‐exposure prophylaxis, PrEP, values and preferences

## Abstract

**Introduction:**

Pre‐exposure prophylaxis (PrEP) is an important HIV prevention option. Two randomized trials have provided efficacy evidence for long‐acting injectable cabotegravir (CAB‐LA) as PrEP. In considering CAB‐LA as an additional PrEP modality for people at substantial risk of HIV, it is important to understand community response to injectable PrEP. We conducted a systematic review of values, preferences and perceptions of acceptability for injectable PrEP to inform global guidance.

**Methods:**

We searched nine databases and conference websites for peer‐reviewed and grey literature (January 2010−September 2021). There were no restrictions on location. A two‐stage review process assessed references against eligibility criteria. Data from included studies were organized by constructs from the Theoretical Framework of Acceptability.

**Results:**

We included 62 unique references. Most studies were observational, cross‐sectional and qualitative. Over half of the studies were conducted in North America. Men who have sex with men were the most researched group. Most studies (57/62) examined injectable PrEP, including hypothetical injectables (55/57) or placebo products (2/57). Six studies examined CAB‐LA specifically. There was overall interest in and often a preference for injectable PrEP, though there was variation within and across groups and regions. Many stakeholders indicated that injectable PrEP could help address adherence challenges associated with daily or on‐demand dosing for oral PrEP and may be a better lifestyle fit for individuals seeking privacy, discretion and infrequent dosing. End‐users reported concerns, including fear of needles, injection site pain and body location, logistical challenges and waning or incomplete protection.

**Discussion:**

Despite an overall preference for injectable PrEP, heterogeneity across groups and regions highlights the importance of enabling end‐users to choose a PrEP modality that supports effective use. Like other products, preference for injectable PrEP may change over time and end‐users may switch between prevention options. There will be a greater understanding of enacted preference as more end‐users are offered anti‐retroviral (ARV)‐containing injectables. Future research should focus on equitable implementation, including real‐time decision‐making and how trained healthcare providers can support choice.

**Conclusions:**

Given overall acceptability, injectable PrEP should be included as part of a menu of prevention options, allowing end‐users to select the modality that suits their preferences, needs and lifestyle.

PROSPERO number: CRD42021285299

## 1 INTRODUCTION

Despite the availability of HIV prevention tools, HIV remains a significant public health issue, with approximately 1.5 million people acquiring HIV in 2021 [1]. Pre‐exposure prophylaxis (PrEP) is the use of antiretroviral drugs to reduce the risk of HIV acquisition among those not infected [2]. In 2021, an estimated 1.6 million people worldwide were using oral PrEP, which was under the United Nations 2025 target of 10 million people [3]. Further complicating the uptake of oral PrEP is the challenge of persistence. To be effective, oral PrEP does not need to be taken continuously, but its impact relies on adherence during periods of risk. Clinical trials have demonstrated that effectiveness is substantially higher among more adherent participants compared to overall study populations [4, 5, 6, 7, 8, 9, 10]. Implementation studies have also demonstrated challenges to the effective use of oral PrEP during periods of risk [11], with uptake and persistence varying across at‐risk populations [12, 13]. Daily pill‐taking can be burdensome for some, including young people [14], contributing to ineffective use.

In January 2021, the dapivirine vaginal ring (DVR) became the second PrEP product recommended by the World Health Organization (WHO) [2]. Although women have expressed interest in the DVR, particularly in east and southern Africa, some have reported discomfort with ring insertion or difficulties with effective use [15]. Newer PrEP products, especially long‐acting modalities, could increase PrEP uptake and use. In 2021, results from two randomized trials on the use of long‐acting injectable cabotegravir (CAB‐LA) as PrEP, HPTN 083 and HPTN 084, were published [16, 17]. These trials stopped early after demonstrating CAB‐LA's statistically superior efficacy at preventing HIV acquisition compared to oral tenofovir/emtricitabine among cisgender men and women and transgender (trans) women. Although CAB‐LA and oral PrEP were effective in both trials, adherence to CAB‐LA was significantly higher than for oral PrEP. In HPTN 084, 93% of women took CAB‐LA as prescribed compared with 42% of women taking oral PrEP [18]. In December 2021, CAB‐LA as PrEP gained approval from the U.S. Food and Drug Administration (FDA) for adolescents and adults at risk for sexual acquisition of HIV [19], with WHO also recommending CAB‐LA as PrEP in July 2022 [20].

Understanding acceptability, interest in and views about injectable PrEP from potential end‐users is key to informing global guidelines to facilitate uptake, thereby increasing coverage of HIV prevention among populations at substantial risk. As such, we conducted a systematic review assessing end‐user and stakeholder values, preferences and perceptions of acceptability related to injectable PrEP. We included peer‐reviewed and grey literature examining all forms of injectable PrEP, including CAB‐LA, placebo injections and hypothetical injectable PrEP use.

## 2 METHODS

This review was conducted in accordance with the Preferred Reporting Items for Systematic Reviews and Meta‐analyses (PRISMA) guidelines [21]. A protocol was reviewed by the WHO and prospectively registered in PROSPERO (CRD42021285299). Database searches and article screening were conducted concurrently with a review on safety and efficacy of CAB‐LA as PrEP (CRD42021290713) [22]. Results are reported separately.

### 2.1 Databases and search terms

In October 2021, we searched for peer‐reviewed and grey literature in databases that sourced clinical or social and behavioural research on the use of injectable PrEP globally. We worked with a reference librarian to construct a search strategy for the following databases: PubMed, Global Health, Cochrane Register of Controlled Trials, Embase and the Cumulative Index to Nursing and Allied Health Literature. Abstracts from the following conferences were searched: International AIDS Conference; International AIDS Society Conference on HIV Pathogenesis, Treatment, and Prevention; Conference on Retroviruses and Opportunistic Infections; and HIV Research for Prevention Conference.

Within each database, we searched for articles published between 1 January 2010 and 27 September 2021. Because we searched for clinical as well as social and behavioural research, we opted for an inclusive search strategy that included three main constructs: injectable modalities AND PrEP/prevention AND HIV. (Supporting Information Appendix A includes a comprehensive list of terms.)

### 2.2 Inclusion and exclusion criteria

Articles needed to meet eligibility criteria (Table 1). We included articles exploring values and preferences for injectable PrEP among diverse stakeholders regardless of language or location of intervention. We excluded articles that did not include primary data, those containing duplicative data or interim results if final results were available.

**Table 1:** Inclusion criteria for the review on values and preferences for injectable pre‐exposure prophylaxis (PrEP)

Criteria	Eligibility
Article type	a. Published in peer‐reviewed journal between 1 January 2010 and 27 September 2021; OR
	b. Presented as abstract at a scientific conference between January 2010 and September 2021; OR
	c. Unpublished work containing relevant data
Intervention	Studies reporting primary data on injectable PrEP, including CAB‐LA, placebo products or hypothetical injectable PrEP use
Study population	a. Populations at substantial risk of HIV acquisition; OR
	b. Healthcare workers/stakeholders involved in any aspect of provision of injectable PrEP
Study design	a. Qualitative studies, including in‐depth interviews or focus group discussions; OR
	b. Experimental or non‐experimental studies quantitatively evaluating the use of injectable PrEP to prevent HIV among people at substantial risk of HIV infection
Key outcomes	a. Awareness of injectable PrEP
	b. Values and preferences related to injectable PrEP
	c. Feasibility^a^, acceptability^b^ or satisfaction^c^ with injectable PrEP
	d. Concerns regarding injectable PrEP
	e. Willingness to use injectable PrEP
	f. Barriers and facilitators of injectable PrEP use

^a^ Feasibility is defined as “the extent to which a new treatment, or an innovation, can be successfully used or carried out within a given agency or setting” [23, 24].

^b^ Acceptability is defined as “a multi‐faceted construct that reflects the extent to which people delivering or receiving a healthcare intervention consider it to be appropriate, based on anticipated or experienced cognitive and emotional responses to the intervention” [25].

^c^ Satisfaction is “the state of being content or fulfilled with a service or intervention based on one's needs and desires or being content with the general service‐delivery experience” [26]. Abbreviations: PrEP, pre‐exposure prophylaxis; CAB‐LA, long‐acting injectable cabotegravir.

### 2.3 Citation screening, data management and analysis

After conducting database searches, a de‐duplicated list of references was uploaded into Covidence, a systematic review screening and data management software. Four reviewers used a multi‐phase screening strategy to determine inclusion (stage 1: title/abstract review; stage 2: full‐text review). Disagreements were resolved by consensus through team discussions. Quantitative and qualitative data were extracted independently by two reviewers using a standardized Excel‐based form. Differences in data extraction were also resolved through consensus. We gathered the following from each included study: (1) study identification: authors, reference type and publication year; (2) description: objectives, location, population characteristics, intervention description, study design and sample size; and (3) outcomes: quantitative or qualitative measures, main findings, strengths, limitations and conclusions. We also coded references to relevant constructs from the Theoretical Framework of Acceptability (TFA; Table 2) [25].

**Table 2:** Constructs of acceptability for injectable PrEP, adapted from the Theoretical Framework of Acceptability developed by Sekhon et al. [25]

Construct	Operationalization	Outcomes
Affective attitude	Overall feelings about an intervention	Satisfaction with, overall acceptability, liking or recommending injectable PrEP
Burden	Perceived amount of effort to participate in the intervention	Ease of use and facilitators; perceived challenges or concerns related to injectable PrEP
Ethicality	Extent of fit with an individual's value system	Discretion of product use; fitting with lifestyle preferences; perceived stigma
Intervention coherence	Extent that participant understands the intervention/how it works	Understanding how injectable PrEP prevents HIV
Opportunity costs	Extent that benefits, profits or values must be given up to engage in the intervention	Trade‐offs of taking injectable PrEP
Perceived effectiveness	Perception that intervention is likely to achieve its purpose	Degree of protection; perceived ability of injectable PrEP to prevent HIV
Self‐efficacy	Participant's confidence that they can perform the behaviours required to participate in the intervention	Ability to use/adhere to injectable PrEP; ability to regularly attend clinic visits to receive the injections

Abbreviation: PrEP, pre‐exposure prophylaxis.

Findings were summarized and reported in narrative and tabular formats. Results are organized by constructs from the TFA. Peer‐reviewed articles were also assessed for risk of bias using the Joanna Briggs Institute (JBI) Critical Appraisal Tools [27], a suite of checklists available by study design. After selecting the appropriate checklist based on each study's design, the research team appraised each article and summarized the results.

## 3 RESULTS

**Figure 1:**
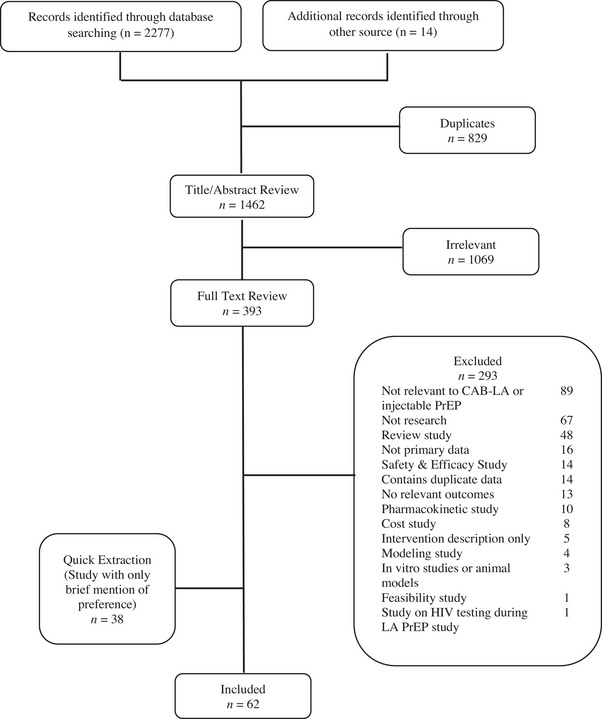
PRISMA diagram.

We identified 2277 records through our database search and 14 records through other sources, yielding 1462 unique records (Figure 1). Ultimately, 100 records met inclusion criteria; 62 records (53 articles and 9 abstracts) were fully extracted and organized below by acceptability constructs and groups, with findings specific to CAB‐LA also reported separately. Thirty‐eight records briefly mentioned preferences for injectable PrEP (e.g. abstracts with few details, articles including only one question mentioning injectable PrEP) and were extracted separately (Supporting Information Appendix B).

### 3.1 Study characteristics

Of 62 records, most were observational, cross‐sectional and qualitative studies examining end‐user values and preferences in approximately 41 countries (one report included participants from 29 unnamed countries), with over 50% of articles from North America and one‐third from sub‐Saharan Africa (SSA; Table 3). Most examined injectable PrEP generally, including hypothetical or placebo injections. Six studies examined CAB‐LA specifically. Men who have sex with men (MSM) were the most researched group. Table 4 summarizes the results from included studies.

**Table 3:** Characteristics of studies included in detailed extraction

Characteristics	*N* of studies (%)
**Study type**	
Peer‐reviewed	53 (85)
Grey literature	9 (15)
**Study design**	
Observational study	58 (94)
Randomized control trial	4 (6)
**Location** **^a^**	
North America	35 (57)
Sub‐Saharan Africa	19 (31)
East Asia and Pacific	5 (8)
Europe and Central Asia	5 (8)
Latin American and the Caribbean	2 (3)
South Asia	2 (3)
**Modality**	
Injectable (generic)	53 (85)
CAB‐LA	6 (10)
Injectable multipurpose prevention technology	2 (3)
Rilpivirine	1 (2)
**Group identifier** **^b^**	
MSM	26 (42)
Women only	11 (18)
Adolescents and young people	8 (13)
Current PrEP users	8 (13)
Trans women and men	6 (10)
Care providers	5 (8)
People who inject drugs	5 (8)
Sex workers (male and female)	5 (8)
**JBI checklist** **^c^**	
Qualitative	27 (51)
Cross‐sectional	22 (41)
Randomized control trial	4 (8)

^a^ A study could include more than one location. Geographic locations were defined according to World Bank Classifications.

^b^ A study could include more than one group identifier.

^c^ Only full‐text peer‐reviewed articles were assessed with a JBI checklist. Abbreviations: PrEP, pre‐exposure prophylaxis; JBI, Joanna Briggs Institute.

**Table 4:** Summary of studies included in detailed extraction

Specific group	First author, et al. (year)	Region	Study design category	Sample size	Product assessed	Main findings	Ref.
**MSM**	Meyers, K. et al. (2014)	North America	Observational study	197	Injectable PrEP (hypothetical products)	Over 80% of participants stated that they would definitely or probably be willing to receive injectable PrEP if it could effectively prevent HIV aquisition. Higher socio‐economic status corresponded with lower willingness to use injectable PrEP (x2 = 5.38, df = 2, *p* = 0.07), as did some college education (x2 = 10.78, df = 2, *p* = 0.004). In bivariate analysis, compared to those with a college or post‐graduate degree, those with some college or technical school had higher odds of being willing to try injectable PrEP (OR = 3.9, 95% CI: 1.7−9.1); however, there was no linear trend across level of education. Those with no partners in the last 3 months had lower odds of being willing to use injectable PrEP as compared to those who reported two or more partners (OR = 0.4, 95% CI: 0.1, 0.9). In the multivariable model, education and number of partners retained significance at *p* < 0.10. 79.2% (*n* = 156, *p* < 0.001) stated they would prefer an injection administered every 3 months. From the bivariate model: if PrEP was accompanied by free sexual health services, one‐on‐one counselling around PrEP use and sexual health, text‐based support, of if it was free, all had significantly higher odds of being willing to use injectable PrEP (ORs ranging from 3.0 to 4.6) than those who disagreed with those statements.	[28]
	Chakrapani, V. et al. (2015)	South Asia	Observational study	26	Injectable PrEP (hypothetical products)	Some MSM reported a preference for injectable PrEP administered monthly or every 2 months over daily oral PrEP. An advantage of injections was that it could solve problems on where to safely store and conceal PrEP at home or how to carry pills during travel.	[29]
	Meyers, K. et al. (2016)	North America	Observational study	62	Injectable PrEP (hypothetical products)	Sixty‐eight percent of participants would “definitely” or “probably” switch to injectable PrEP if it were FDA approved. Product‐specific motivating factors for “switchers” included: convenience, not having to adhere to daily pill and not carrying pills around. Psychological factors included desire to eliminate anxiety around missed dosing and a belief that a shot would mean “less to think/worry about.” Those not willing to switch from oral to injectable PrEP cited scientific concerns, including safety, efficacy and waning protection. Product‐level disadvantages included pain and inconvenience of scheduling follow‐up injections. Non‐switchers reported that swallowing a pill daily made them feel like they were in control of their health and would not trust the shot's protection. They expressed satisfaction with oral PrEP and unwillingness to be an early adopter of a novel approach.	[30]
	Oldenburg, C. E. et al. (2016)	East Asia and Pacific	Observational study	548	Injectable PrEP (hypothetical products)	Reasons for preferring injectables included: easier to remember than a daily pill (71%) and easier to conceal from members of their community (58.1%). Older participants had increased odds of preferring injectable PrEP (aOR 1.08 per 1‐year increase in age, 95% CI 1.03−1.14). Participants who were willing to pay more for PrEP had increased odds of preferring injectable PrEP (aOR 1.17 per 1‐unit increase in amount of willing to pay for PrEP, 95% CI 1.01−1.35).	[31]
	Parsons, J. T. et al. (2016)	North America	Observational study	948	Injectable PrEP (hypothetical products)	When asked about the acceptability of injectable PrEP in 1‐ and 3‐month intervals, 43.2% found the 1‐month version acceptable compared with 53.6% who found the 3‐month dosing acceptable. Forty‐six percent expressed a preference for injectable PrEP (vs. 14.3% for oral PrEP and 21.7% for whichever turns out to be most effective). Those who would not take PrEP at all were significantly older (*p* < 0.05) than those who preferred injectable PrEP and those who would take the most effective form. Those who would take the most effective form were also significantly younger than those who preferred oral PrEP. Long‐term health effects and concerns about side effects were the greatest concern for both oral and injectable PrEP. Participants reported having to return for medical visits every 3 months as significantly less of a burden for injectable PrEP than oral PrEP.	[32]
	Greene, G. J. et al. (2017)	North America	Observational study	512	Injectable PrEP (hypothetical products)	Among PrEP modalities, the most preferred was the implant‐1 (21.5%; two implants; 12‐month duration; tactile, but not visible perception by others), followed by daily oral PrEP (17%), injectable PrEP (14.3%) and implant‐2 (3.7%; two implants; 12‐month duration; tactile and visible perception). There was higher educational attainment reported by those preferring injections over those preferring condoms and pill (*p* = 0.04 and *p* = 0.02, respectively). For those who preferred injections, frequently cited reasons were protection duration (49.5%), dislike/fear of other options (49.5%) and privacy (32.1%).	[33]
	Ngongo, P. B. et al. (2017)	Africa	Observational study	165	Injectable PrEP (hypothetical products)	There was a difference of opinion on mode of administration, with healthcare providers and MSM preferring oral PrEP and other groups (FSW, youth) opting for injectable PrEP.	[34]
	Beymer, M. et al. (2018)	North America	Observational study	761	Injectable PrEP (hypothetical products)	Nearly, 75% of the sample indicated they would be more willing to try injectable PrEP than daily oral PrEP. Those who had some college education or more had higher odds of willingness to try injectable PrEP (aOR = 2.92; 95% CI: 1.32−6.46).	[35]
	Biello, K. B. et al. (2018)	North America	Observational study	36	Injectable PrEP (hypothetical products)	Participants with prior PrEP experience expressed greater preference for daily pills as a trusted method and were uncertain about injectable dose and side effects. PrEP‐naïve participants expressed greater interest in injectables due to longer intervals between doses. Participants across groups agreed that injectable PrEP might be preferred for certain people/circumstances. Quarterly injections may be more manageable for those with adherence difficulties, those who engage in sex more frequently and may avoid stigma associated with PrEP use (more discreet). Concerns about injectable PrEP were related to having to return for an injection every 3 months, missing medical appointments, diminishing levels of protection, experience of getting an injection and side effects.	[36]
	Calder, B. J. et al. (2018)	North America	Observational study	21	Injectable PrEP (hypothetical products)	MSM expressed that adherence to pill‐taking was sufficient to ensure a high level of protection against HIV. Improved adherence associated with the usage of long‐acting modalities (injections and implants) was not an advantage. Some MSM saw value in injections and implants based on the belief that it could simplify their busy lives, but they were unsure if potential benefits would outweigh the disadvantages (e.g. having less control). Although MSM saw oral PrEP as already effective, they would value long‐acting modalities if they were more effective and less costly.	[37]
	Dubov, A. et al. (2018)	Europe and Central Asia	Observational study	1184	Injectable PrEP (hypothetical products)	In a choice‐based analysis, members of a group that had more students, live off a low income, well‐educated (university level) (*n* = 216, 18%) had a strong preference for injectable PrEP. Most were influenced by cost.	[38]
	John, S. A. et al. (2018)	North America	Observational study	104	Injectable PrEP (hypothetical products)	30.8% of the men specifically preferred injectable PrEP, and 34.6% preferred the most effective method. Participants had the lowest amount of concern regarding fear/dislike of needles associated with injectable PrEP (M = 1.75, SD = 1.10, range 1–4), with the majority (61.5%) having no concern at all about fear/dislike of needles. Men had less concern about returning for medical check‐ups and injections every 3 months (M = 1.79, SD = 1.00, range 1–4), with 54.8% having no concern about quarterly medical visits. Men reported moderate concern regarding long‐term health effects of injectable PrEP (M = 2.63, SD = 0.97, range 1–4). Most had a moderate amount of concern about the potential side effects of injectable PrEP (M = 2.71, SD = 0.91, range 1–4). Nearly, all (93.3%) expressed some level of concern about incomplete protection, and 40.4% of all men were very concerned about it. In bivariate analyses, men with less than a bachelor's degree had higher odds of preferring injectable PrEP compared to those with more education. Concerns about incomplete HIV protection and possibility of protection wearing off were both associated with lower odds of injectable PrEP preference.	[39]
	Kerrigan, D. et al. (2018)	North America	Observational study	26	CAB‐LA (enacted preference)	Significant consensus that while the injections were “not always pleasant,” side effects were worth the pain if injectable PrEP was found to be effective. Most participants rated their satisfaction as “very high” and almost all indicated their interest in potentially using CAB‐LA. The main disadvantage of injectables: injection site reactions, large needle size and exposing one's buttocks to receive the injections. Participants described receiving injections every 3 months as convenient. There was a perceived advantage over not having to worry about adhering to a daily oral regimen. CAB‐LA afforded more confidentiality and privacy than daily oral pills.	[40]
	Meyers, K. et al. (2018)	North America	Observational study	28	CAB‐LA (enacted preference)	Eighty‐eight percent (*n* = 14) reported they would definitely or very likely use CAB‐LA, and 63% (*n* = 10) reported they would prefer to use CAB‐LA every 12 weeks to daily oral PrEP. Sixty‐four percent (*n* = 20) felt anxiety before the first injection. This decreased to 29% (*n* = 8) by the second and third injections. Participants reported two sources of anxiety: needles (32%, *n* = 9) and expectation of injection pain (54%, *n* = 15). Some expressed feelings of awkwardness and vulnerability associated with receiving injections in the buttocks (43%, *n* = 12). Many participants (46%, *n* = 13) spoke about the importance of nurses and staff being caring, friendly and warm, and treating them like human beings rather than just study subjects. They also discussed the importance of providers managing patients’ expectations and providing information about the process. Fourteen percent (4/28) of participants mentioned the importance of the skill‐level of study nurses in terms of drawing blood and giving injections and the fact that they liked having the same person each time.	[41]
	Meyers, K. et al. (2018)	East Asia and Pacific	Observational study	200	Injectable PrEP (hypothetical products)	Seventy‐six percent (152/200) of the men said they would be probably or very willing to use injectable PrEP. In multivariate analyses, higher education (aOR = 0.5, 95% CI 0.2−1.0), having a female partner (aOR = 3.1, 95% CI 1.0, 10.2), knowing a person living with HIV (aOR = 4.2, 95% CI 1.9, 9.2) and having a steady partner living with HIV (aOR = 0.0, 95% CI 0.0, 0.3) were significant predictors for having interest in injectable PrEP only. Ninety‐two (46.0%) respondents were unwilling to consider oral PrEP. Of these 92, 53.3% (49/92) would consider an injectable PrEP modality. Of the men who found oral PrEP acceptable, 95.4% (103/108) were also open to injectable PrEP.	[42]
	Murray, M. I. et al. (2018)	North America	Randomized control trial	115	CAB‐LA (enacted preference)	At week 30, participants were generally satisfied with the study medication (100% in placebo, 74% in CAB‐LA). Overall, participants reported a willingness to recommend the treatment (CAB‐LA, 87%, placebo, 100%) and continue the study medication (CAB‐LA, 79% and placebo, 100%). Tolerability assessments of cabotegravir injections showed that 66% (*n* = 57) of participants were satisfied with the side effects and 64% (*n* = 55) were satisfied with the amount of pain/discomfort. Throughout the study, 79% of participants in the cabotegravir group reported a level of satisfaction sufficient to continue with the study medication (Week 6, 88%; Week 18, 81%; Week 30, 79%). When comparing views of treatment at Week 18, participants were more satisfied with injectable cabotegravir than with oral cabotegravir, particularly on items related to convenience, flexibility and lifestyle. Approximately 25% of participants in the cabotegravir treatment group reported dissatisfaction with the amount of discomfort or pain associated with the study medication during the injection phase. However, 74% (*n* = 67/91) of participants were satisfied to continue treatment with injectable cabotegravir, and few (4%; *n* = 4/94) withdrew from the study because they reported injection intolerability.	[43]
	Patel, R. R. et al. (2018)	North America	Observational study	26	Injectable PrEP (hypothetical products)	17/26 MSM preferred injectable PrEP, regardless of frequency. Some respondents did not want an injection without discussing frequency. Six participants mentioned that who administers the injection was tied to their preferences for an injectable modality. Seven participants reported fear of needles when discussing preferences for injections versus other modalities. When asked about acceptability for injectable PrEP (administered every 3 months), most interviewees (18/22) found injectable PrEP acceptable. Acceptability was related to familiarity with other medications that are in an injectable form, infrequent dosing, disliking injections and knowledge of efficacy. Participants’ preferences for injections factored in general dislike for needles, the frequency that they needed to receive an injection and not wanting to self‐administer the injection. Preferred frequencies regarding injectable PrEP typically ranged from weekly to annually. Longer dosing intervals were proposed and preferred (3 months)	[44]
	Dubov, A et al. (2019)	Europe and Central Asia	Observational study	554	Injectable PrEP (hypothetical products)	Results from discrete choice experiment showed that Group 1 (*n* = 73)—described as average age of 41.2, 78% work full time, 96% has associate degree or higher, 86% White, 75% live in urban area—had equal preference for either daily pill or on‐demand intermittent pills while strongly opposing injectable PrEP. Group 2 (*n* = 126)—described as 72% working full time, 96% had an associate degree or higher, 86% White, 71% living in urban areas—put cost twice as important as dosing frequency, and preferred injectable PrEP. Those preferring injectable PrEP are overall younger and better educated.	[45]
	Ellison, J. et al. (2019)	North America	Observational study	108	Injectable PrEP (hypothetical products)	In ranking the likelihood of using products, subdermal implants were most commonly selected as the first choice (45%), followed by injectables (31%) and daily oral PrEP (21%). Among those who were somewhat or very interested in injectables, the most common reasons were: not having to take a daily pill (43%), convenience (30%) and timing or dosage frequency (11%). Regarding the most important reasons for not wanting to use injectable PrEP, participants who were not very or not at all interested cited disliking needles (46%), concerns about safety or effectiveness (23%) and logistical difficulties (15%). In multivariate analyses, Black and Hispanic MSM were more likely (OR: 2.45, 95% CI: 0.86−6.89) to prefer the injectable over daily oral PrEP. MSM with public insurance also had increased odds ([aOR]: 2.80, 95% CI: 0.71−11.1) of preferring injectable PrEP.	[46]
	Peng, L. et al. (2019)	East Asia and Pacific	Observational study	524	Injectable PrEP (hypothetical products)	Overall willingness to use any type of PrEP in the next 6 months was 84.9%, with a willingness rate of 60.1% for daily oral PrEP, 79.2% for on‐demand PrEP and 62.8% for injectable PrEP. Most participants intended to adhere to PrEP, with an intention rate of 70.2% for daily PrEP, 84.9% for on‐demand PrEP and 71.2% for injectable.	[47]
	Minnis, A. M. et al. (2020)	Africa	Observational study	807	Injectable PrEP (hypothetical products)	Females and MSM both had greater preference for a single injection over an implant compared to MSW (*p* = < 0.004). Females and MSW also expressed more preference for two injections compared with implants (*p* < = 0.009). All youth preferred insertion in the arm (*p*<0.001). Females disliked insertion in the thigh, and both MSW and MSM disliked insertion on the buttocks (*p* = 0.01). If a clinic offered a 2‐month long‐acting PrEP product, females would be willing to go to a pharmacy if the pharmacy offered a product dosed every 3.8 months (95% CI 3.0, 5.5); if the clinic offered a 6‐month long‐acting PrEP product, they would be willing to go to the pharmacy if it offered a product dosed every 9 months (95% CI 7.7, 10.4).	[48]
	Torres, T. et al. (2020)	Latin American and the Caribbean	Observational study	19,457	Injectable PrEP (hypothetical products)	Overall, injectable PrEP was the first option for 42% (95% CI 41–43) of the respondents. In multivariable logistic regression, preference for injectable PrEP was associated with age >25 (aOR 1.32, 95% 1.22−1.44), PrEP awareness (aOR 1.23, 95% CI 1.15−1.32), having >5 male sexual partners (aOR 1.05, 95% CI: 0.99−1.12) and binge drinking (aOR 1.15, 95% CI 1:07−1.23)	[49]
	Gutierrez, J. I. et al. (2021)	North America	Observational study	429	Injectable PrEP (hypothetical products)	An injectable PrEP option did not alter participant interest rate among PrEP‐experienced individuals but did garner a higher participation interest rate in those with no PrEP experience (80.5%).	[50]
	Macapagal, K. et al. (2021)	North America	Observational study	59	Injectable PrEP (hypothetical products)	Participants disliked how injections and removable implants require more frequent visits to healthcare providers and require greater commitment. Participants liked when delivery methods were less perceptible or visible (e.g. injectable). Participants most frequently discussed disliking the possibility of side effects (i.e. pain and scarring at the insertion or injection site). Barriers to injectables: dislike of injection and frequency of the injection (every 2 months). To increase acceptability, participants suggested administration in their arm instead of buttocks, self‐administration like some gender‐affirming hormones and reduced injection frequency. When asked to rank order preferred methods, most youth selected condoms (39.6%) and yearly implants (37.7%)—injection and quarterly implant were the least preferred.	[51]
	Mansergh, G. et al. (2021)	North America	Observational study	782	Injectable PrEP (hypothetical products)	Most men were likely to use PrEP in the future via injection (74%). In multivariable analysis, current PrEP users (vs. non‐users) were more likely to prefer injectable (aOR = 3.29, 95% CI = 2.12−5.11). Black (vs. White) men had lower odds for reporting likelihood of using injectable (AOR = 0.54, 95% CI = 0.34−0.84). MSM age 30−39 (vs. older men) had greater odds of endorsing an injection product (aOR = 1.89, 95% CI = 1.17−3.07). Current daily PrEP users (vs. non‐users) ranked injectable and daily oral PrEP higher, and condoms, event‐based pills, anal gel and anal suppositories were ranked lower. White MSM ranked injectables higher than Black and other or mixed race/ethnicity men, but not compared to Hispanic/Latino men.	[52]
	Nguyen, L. H. et al. (2021)	East Asia and Pacific	Observational study	30	Injectable PrEP (hypothetical products)	One participant suggested that the injectable PrEP should be provided along with oral medication. It could be beneficial for the PrEP users as they did not need to remember taking the pill daily, which could increase uptake.	[53]
**Women**							
	Luecke, E. H. et al. (2016)	Africa	Observational study	68	Injectable PrEP (hypothetical products)	Most women (55 of 68, 81%) preferred long‐acting formulations. Women who did not complete secondary school were significantly less likely to prefer long‐acting methods (70% vs. 94%, *p* < 0.05). Women were very concerned about their ability to adhere to daily regimens and often expressed a preference for product formulations that are long‐acting or on demand. Product formulations administered at the clinic by providers were also attractive because women would not have store or hide products at home.	[54]
	van der Straten, A. et al. (2017)	Africa	Observational study	71	Injectable PrEP (hypothetical products)	This study was conducted within ASPIRE, a phase III randomized controlled trial of a vaginal ring, with all women being ring experienced. The vaginal ring was most preferred by 94% of participants, followed by implants (39%), injectables (33%), male condoms (27%) and oral tablets (11%). Product reversibility came up in discussions of safety and side effects. The potential for incompatibility between the drug and the body was seen as a major disadvantage of injections. Preference for long‐acting formulations was also explained by an appreciation for infrequent dosing, fewer clinic visits and less opportunities to forget doses, the ease of administering the product at the clinic, discreetness, and in the case of the implant and injection, non‐vaginal administration. Concerns with injections were: needle phobia, the pain or discomfort associated with the administration process, the reliance on skilled clinicians and the invasive procedure.	[55]
	Minnis, A. M. et al. (2018)	Africa	Randomized control trial	258	MPT injections (placebo products)	Product ratings after 1 month of use were highest for injections (mean 4.26; 95% CI 4.14, 4.38). The mean rating for injections was significantly higher than those for rings and tablets (*p* <0.001); the mean rating for rings was also significantly higher than that for tablets (*p* = 0.015). For women who expressed negative reactions to the injection, they discussed being afraid of pain with the injections when they first saw the needle; however, these feelings subsided after receiving them. Participants who had completed secondary school reported a lower mean rating for tablets compared with those who had not completed secondary school (adjusted β −0.38; 95% CI −0.71, −0.06; *p* = 0.021), and a higher mean rating for injections (adjusted β 0.24, 95% CI 0.00, 0.49, *p* = 0.054). Ever‐use of injectable contraceptives was associated with slightly lower injection ratings (adjusted β −0.24, 95% CI −0.48, 0.00, *p* = 0.054). For injections, the level of acceptability of product‐specific attributes was higher than for the other products. Women who indicated the injection's attributes tied to use were acceptable very consistently expressed positive views of the product, valuing the fact that it “saved time” and offered discreetness.	[56]
	Quaife, M. et al. (2018)	Africa	Observational study	609	Injectable PrEP (hypothetical products)	%1.Adult women and FSWs significantly disliked oral PrEP and favoured injectable products. Neither adult women nor adolescent girls found the vaginal ring appealing but an injectable product was favoured by all groups.	[57]
	Minnis, A. M. et al. (2019)	Africa	Randomized control trial	523	MPT injections (placebo products)	Thirty‐four percent of women who chose the injection after trying each of the three products (*N* = 160) had preferred another product at enrolment (25% tablets, 9% rings). The injection achieved the highest adherence, which was also the most popular delivery form. Oral pills achieved the lowest adherence. Although injections were the favoured delivery form, discrete choice experiment data estimated that a larger proportion of participants would choose an MPT ring or MPT tablets over injections that prevented only HIV.	[58]
	Tolley, E. E. et al. (2019)	North American, Africa	Observational study	136	Rilpivirine (enacted preference)	Although U.S. participants reported lower acceptability of injectable attributes than women from African sites, most participants rated injectable attributes as highly acceptable. Acceptability ratings were similar over time. At baseline, 56% of U.S. participants and 81% of African participants preferred using a bi‐monthly injectable to other prevention methods, including daily oral pills, a vaginal ring or gel. Interest increased in both regions over time. At week 28, 79% of participants strongly endorsed that they would “definitely use an injectable PrEP product for some time” if it were available in the future. Even more women (88%) strongly agreed that they would be “more interested in using an injectable if it was both for HIV and pregnancy prevention.” In longitudinal univariate models, acceptability of product attributes and of physical experiences, altruistic and personal motivations for trial participation, and HIV risk perception, were associated with future interest in use. U.S. participants reported significantly lower levels of future interest in use than African women (OR = 0.14, *p* < 0.001) and those in the experimental group had lower future interest in use than those in the placebo arm (OR = 0.42, *p* = 0.04). Product attribute scores and being from a non‐U.S. site remained the strongest predictors of future interest in use in multivariate models.	[59]
	Calabrese, S. K. et al. (2020)	North America	Observational study	563	Injectable PrEP (hypothetical products)	The strongest preference was for daily pills (29.4%) and injections (24.3%). In multivariable analyses, women currently using injectable contraceptive method had higher odds of preferring PrEP injections than those never having used injectable contraceptives (aOR 8.45, 95% CI 4.22−16.91).	[13]
	Dauria, E. F. et al. (2021)	North America	Observational study	27	Injectable PrEP (hypothetical products)	Criminal justice‐involved women expressed most interest in oral and injectable versions of PrEP due to familiarity with medication administered in this way.	[60]
	Philbin, M. M. et al. (2021)	North America	Observational study	59	Injectable PrEP (hypothetical products)	Fifty percent of women would prefer injectable PrEP over daily pills. Many women compared HIV therapy to birth control options—these individuals focused primarily on how experience with injections would allow people to feel comfortable with future injections and did not feel that taking multiple injections at once would be a barrier. Women with a history of frequent medication‐related injection (e.g. insulin pumps, etc.) expressed reticence to add additional injectables to their regimen. Many women reported being terrified of needles and explained that they would not use injectable formulations no matter how much they disliked pills. Among women who had history of injecting drugs, some stressed that they would avoid injectables because even the sight of a needle “can be a trigger,” but some women with a history of drug use described how it would be an individual‐level decision about whether a history of injection drug use might limit their willingness to use injectables. Most women felt that people who currently inject drugs would be especially willing to try injectable modality for treatment and prevention.	[61]
	Philbin, M. M. et al. (2021)	North America	Observational study	30	Injectable PrEP (hypothetical products)	Specific barriers to injectable PrEP uptake included medical mistrust (e.g. women expressed a desire to wait until injectable PrEP had been on the market for an extended period to ensure its safety and efficacy), injection‐related side effects (e.g. interfering with a pregnancy), administration location and more frequent doctors’ visit. Facilitators included belief that shots were more effective than pills, convenience and confidentiality.	[62]
	Tolley, E. et al. (2022)	Africa	Observational study	68	CAB‐LA (enacted preference)	Participants overwhelmingly preferred CAB LA to daily pills. Regardless of risk category, women liked the injectable's privacy from husbands, boyfriends, sexual clients or just “nosey people.” At least half of participants worried about forgetting to take pills, describing previous mishaps with oral contraception or challenges with study pills. Descriptions of pain were variable, but the most common concern with the injection. Women in high‐risk categories were more likely to mention “effectiveness” as a reason to prefer the injection.	[63]
**Adolescents and young people**
	Mack, N. et al. (2014)	Africa	Observational study	133	Injectable PrEP (hypothetical products)	In South Africa, adolescents’ concerns about injections included the potential to be painful, with some preferring an injection in the arm rather than buttocks. Several girls were uncomfortable with having to remove their pants/skirts to receive an injection. Young women preferred injections over a pill because they thought injections would be safer, longer‐lasting, more private and difficult to forget. Young women expressed less concerns over receiving an injection in the buttocks—some actually preferred that location since they were already accustomed to receiving contraceptive injections there.	[64]
	Ngongo, P. B. et al. (2017)	Africa	Observational study	165	Injectable PrEP (hypothetical products)	There was a difference of opinion on mode of administration, with healthcare providers and MSM preferring oral PrEP and other groups (FSW, youth) opting for injectable PrEP.	[34]
	Quaife, M. et al. (2018)	Africa	Observational study	609	Injectable PrEP (hypothetical products)	Adult women and FSWs significantly disliked oral PrEP and favoured injectable products. Neither adult women nor adolescent girls found the vaginal ring appealing but an injectable product was favoured by all groups.	[57]
	Montgomery, E. T. et al. (2019)	Africa	Observational study	95	Injectable PrEP (hypothetical products)	Injections could address the issue of the ring potentially come out and causing embarrassment. They could allow for greater sexual freedom and pleasure. Injectable‐experienced women appreciated not having a daily product and felt that receiving the injection every 2–3 months was feasible. The injection was perceived to provide greater protection because it would be absorbed in their bodies, they would not forget to take it and it would not burst. Experience of side effects was raised as the main complaint with using injectable PrEP. Some stated that injection site pain was also a disadvantage. For some, high efficacy would override the importance of all other attributes. Assuming high efficacy, the most favourable attribute among HIV prevention methods was invisibility, linked to long‐acting modalities.	[65]
	Taggart, T. et al. (2019)	North America	Observational study	200	Injectable PrEP (hypothetical products)	Thirty‐four percent said they prefer daily oral PrEP, 13% prefer PrEP injections and 14.7% prefer a PrEP implant. Reasons for preferring PrEP injections over other modalities included: “long lasting” and “would not have to take a pill everyday.”	[66]
	Golub, S. A. et al. (2020)	North America	Observational study	93	Injectable PrEP (hypothetical products)	Young people want to understand how biomedical prevention products are going to protect them. For injectable PrEP, young people had questions about why the shot needed to be “in the butt,” what the shot feels like and whether there is soreness after. Participants raised questions about the feasibility of injectable PrEP in the gluteus for those receiving gluteal silicone injections. For some, this was seen as a barrier for use, but participants immediately thought creatively about marketing long‐acting gluteal injections as part of gender‐affirming care.	[67]
	Kidman, R. et al. (2020)	Africa	Observational study	2085	Injectable PrEP (hypothetical products)	Over 80% of both genders would be willing to have an injection every 3 months. Both genders preferred to get an injection at a health clinic (59−65%) over taking a daily pill. When the alternative was giving themselves an injection at home, approximately half favoured an injection over a daily pill. One‐quarter of participants had concerns that might stop them from getting an injection: that it might make them sick (6%), privacy (5%) and they may forget to get it (4%). Over 10% said that they had a fear of injections/needles or were concerned about the pain.	[68]
	Minnis, A. M. et al. (2020)	Africa	Observational study	807	Injectable PrEP (hypothetical products)	Females youth expressed more preference for two injections compared with implants (*p* < = 0.009). All youth preferred insertion in the arm (*p*<0.001). Females disliked insertion in the thigh. If a clinic offered a 2‐month long‐acting PrEP product, females would be willing to go to a pharmacy if the pharmacy offered a product dosed every 3.8 months (95% CI 3.0, 5.5); if the clinic offered a 6‐month long‐acting PrEP product, they would be willing to go to the pharmacy if it offered a product dosed every 9 months (95% CI 7.7, 10.4).	[48]
**PrEP users**							
	Meyers, K. et al. (2016)	North America	Observational study	62	Injectable PrEP (hypothetical products)	Sixty‐eight percent of participants would “definitely” or “probably” switch to injectable PrEP if it were FDA approved. Product‐specific motivating factors for “switchers” included: convenience, not having to adhere to daily pill and not carrying pills around. Psychological factors included desire to eliminate anxiety around missed dosing and a belief that a shot would mean “less to think/worry about.” Those not willing to switch from oral to injectable PrEP cited scientific concerns, including safety, efficacy and waning protection. Product‐level disadvantages included pain and inconvenience of scheduling follow‐up injections. Non‐switchers reported that swallowing a pill daily made them feel like they were in control of their health and would not trust the shot's protection. They expressed satisfaction with oral PrEP and unwillingness to be an early adopter of a novel approach.	[30]
	John, S. A. et al. (2018)	North America	Observational study	104	Injectable PrEP (hypothetical products)	30.8% of the men specifically preferred injectable PrEP, and 34.6% preferred the most effective method. Participants had the lowest amount of concern regarding fear/dislike of needles associated with injectable PrEP (M = 1.75, SD = 1.10, range 1–4), with the majority (61.5%) having no concern at all about fear/dislike of needles. Men had less concern about returning for medical check‐ups and injections every 3 months (M = 1.79, SD = 1.00, range 1–4), with 54.8% having no concern about quarterly medical visits. Men reported moderate concern regarding long‐term health effects of injectable PrEP (M = 2.63, SD = 0.97, range 1–4). Most had a moderate amount of concern about the potential side effects of injectable PrEP (M = 2.71, SD = 0.91, range 1–4). Nearly, all (93.3%) expressed some level of concern about incomplete protection, and 40.4% of all men were very concerned about it.	[39]
						6. In bivariate analyses, men with less than a bachelor's degree had higher odds of preferring injectable PrEP compared to those with more education. Concerns about incomplete HIV protection and possibility of protection wearing off were both associated with lower odds of injectable PrEP preference.	
	Meyers, K. et al. (2018)	North America	Observational study	28	CAB‐LA (enacted preference)	Eighty‐eight percent (*n* = 14) reported they would definitely or very likely use CAB‐LA, and 63% (*n* = 10) reported they would prefer to use CAB‐LA every 12 weeks to daily oral PrEP. Sixty‐four percent (*n* = 20) felt anxiety before the first injection. This decreased to 29% (*n* = 8) by the second and third injections. Participants reported two sources of anxiety: needles (32%, *n* = 9) and expectation of injection pain (54%, *n* = 15). Some expressed feelings of awkwardness and vulnerability associated with receiving injections in the buttocks (43%, *n* = 12). Many participants (46%, *n* = 13) spoke about the importance of nurses and staff being caring, friendly and warm, and treating them like human beings rather than just study subjects. They also discussed the importance of providers managing patients’ expectations and providing information about the process. Fourteen percent (4/28) of participants mentioned the importance of the skill‐level of study nurses in terms of drawing blood and giving injections and the fact that they liked having the same person each time.	[41]
	Meyers, K. et al. (2018)	North America	Observational study	105	Injectable PrEP (hypothetical products)	Two‐thirds of participants reported they would definitely (36.2%) or probably (30.5%) switch from daily oral PrEP to injectable PrEP. Those with moderate income ($30,000−$50,000) were more likely to consider switching, compared to those who reported higher income. Respondents who identified as having high injection tolerance had higher odds of switching (OR = 1.64); those who were more concerned about post‐injection pain had lower odds of switching (OR = 0.57). Participants who felt more strongly that the injection interval was a disadvantaged of the shot had lower odds of intending to switch (OR = 0.30). However, some switchers mentioned that oral PrEP requires regular clinic visits anyway, so that regular visits for injections were not an additional burden. Strong agreement with the statement “taking PrEP pills every day feels like an emotional burden” was associated with intention to switch (OR = 1.54). Assigning a higher score to the statement “Taking PrEP pills every day makes me feel responsible” (OR = 1.87) was associated with higher odds of switching. Higher scores on the statement “Not taking a pill every day would make me feel less in control of my HIV prevention” were associated with lower odds of switching (OR = 0.55). Participants expressed concerns about waning protection across the injection interval. In multivariable analysis, two predictors negatively predicted switch intentions: negative ratings of product‐level factors (injections interval [aOR = 0.37] and shot schedule [aOR = 0.31]), three psychosocial‐level factors positively predicted switch intentions: identifying daily pill‐taking as emotionally burdensome (aOR = 13.71), believing that PrEP signifies personal responsibility (aOR = 4.72) and self‐identifying as an early adopter (aOR = 3.77).	[69]
	Ellison, J. et al. (2019)	North America	Observational study	108	Injectable PrEP (hypothetical products)	In ranking the likelihood of using products, subdermal implants were most commonly selected as the first choice (45%), followed by injectables (31%) and daily oral PrEP (21%). Among those who were somewhat or very interested in injectables, the most common reasons were: not having to take a daily pill (43%), convenience (30%) and timing or dosage frequency (11%). Regarding the most important reasons for not wanting to use injectable PrEP, participants who were not very or not at all interested cited disliking needles (46%), concerns about safety or effectiveness (23%) and logistical difficulties (15%). In multivariate analyses, Black and Hispanic MSM were more likely (OR: 2.45, 95% CI: 0.86−6.89) to prefer the injectable over daily oral PrEP. MSM with public insurance also had increased odds ([aOR]: 2.80, 95% CI: 0.71−11.1) of preferring injectable PrEP.	[46]
	Montgomery, E. T. et al. (2019)	Africa	Observational study	95	Injectable PrEP (hypothetical products)	Injections could address the issue of the ring potentially come out and causing embarrassment. They could allow for greater sexual freedom and pleasure. Injectable‐experienced women appreciated not having a daily product and felt that receiving the injection every 2–3 months was feasible. The injection was perceived to provider greater protection because it would be absorbed in their bodies, they would not forget to take it and it would not burst. Experience of side effects was raised as the main complaint with using injectable PrEP. Some stated that injection site pain was also a disadvantage. For some, high efficacy would override the importance of all other attributes. Assuming high efficacy, the most favourable attribute among HIV prevention methods was invisibility, linked to long‐acting modalities.	[65]
	Slama, L. et al. (2019)	Europe and Central Asia	Observational study	200	Injectable PrEP (hypothetical products)	PrEP users were significantly more likely than people living with HIV to mention being certain not to forget treatment (*p* < 0.0001) and no need to think of treatment every day (*p* < 0.0001) as perceived advantages. PrEP users were more likely than PLWH to select loss of freedom (*p* < 0.0001), misgiving adverse effects (*p* = 0.013) as perceived drawbacks and less likely to select too much a constraint as perceived drawback (*p* < 0.0001).	[70]
	Carillon, S. et al. (2020)	Europe and Central Asia	Observational study	28	Injectable PrEP (hypothetical products)	The management of injectable PrEP was perceived both as apprehension and a simplification of patient's daily life. For current daily oral PrEP users, changing treatment to injectable PrEP did not cause apprehension. Some participants dreaded injection as a delivery mechanism.	[71]
**Transgender men and women**
	Meyers, K. et al. (2018)	North America	Observational study	28	CAB‐LA (enacted preference)	Eighty‐eight percent (*n* = 14) reported they would definitely or very likely use CAB‐LA, and 63% (*n* = 10) reported they would prefer to use CAB‐LA every 12 weeks to daily oral PrEP. Sixty‐four percent (*n* = 20) felt anxiety before the first injection. This decreased to 29% (*n* = 8) by the second and third injections. Participants reported two sources of anxiety: needles (32%, *n* = 9) and expectation of injection pain (54%, *n* = 15). Some expressed feelings of awkwardness and vulnerability associated with receiving injections in the buttocks (43%, *n* = 12). Many participants (46%, *n* = 13) spoke about the importance of nurses and staff being caring, friendly and warm, and treating them like human beings rather than just study subjects. They also discussed the importance of providers managing patients’ expectations and providing information about the process. Fourteen percent (4/28) of participants mentioned the importance of the skill‐level of study nurses in terms of drawing blood and giving injections and the fact that they liked having the same person each time.	[41]
	Rael, C. T. et al. (2020)	North America	Observational study	19	Injectable PrEP (hypothetical products)	Participants were familiar with injections, since many have already administered gender‐affirming hormones in this way and were open to receiving injectable PrEP. Participants liked that they would not have to adhere to a daily product. Participants worried that the medication contained in injections and implants could interact with gender‐affirming hormones. Participants felt that clinical studies should examine the effects prevention strategies have on transgender women. Some participants expressed concerns over injection in the gluteal muscles. Participants felt that visits with their healthcare provider to administer injectable PrEP were cumbersome and inconvenient. Transgender women often already juggle multiple doctor's appointments. Other participants felt that intramuscular injections were something they could do at home. The 30‐day oral lead in required for the injection was unpopular among most participants. Some participants were concerned about potential side effects of the medication contained in injectable PrEP. Overall, participants were excited about the concept of long‐acting HIV prevention strategies. Some felt that 6 months was reasonable, though this was the least amount of time anybody found acceptable.	[72]
	Appenroth, M. et al. (2021)	North America, Africa, East Asia and Pacific, Europe and Central Asia, Latin American	Observational study	50	Injectable PrEP (hypothetical products)	Despite some concern, injectable PrEP was most referred to as the preferred modality because it limits the chance of forgetting to take the daily medication and reduces the risk for trans people of carrying around drugs that might expose them as vulnerable to HIV or even being falsely assumed of being HIV positive in front of authorities. Having to go back to see a medical provider regularly to receive the shots was as a limiting factor.	[73]
	Ashodaya Samithi (2021)	South Asia	Observational study	165	CAB‐LA (hypothetical products)	Most community members were excited about CAB‐LA, particularly male sex workers and trans women sex workers. However, there were queries about CAB‐LA side effects, HIV resistance, adherence support, and so on. Trans women sex workers expressed that CAB‐LA would be a good option if it does not interfere with gender‐affirming hormones. Some expressed worry about the pain on the injection site. Though many MSW and trans women sex workers were interested in CAB‐LA, some were sceptical and would prefer to wait and see its use in India before they start taking it for themselves. Many FSWs relate CAB‐LA with Depo‐Provera injection. The long‐acting prevention tool would relieve them from the worry of getting HIV and would ensure the daily oral tablet is not required. The primary concern regarding taking the injection from government hospitals remained long waiting times, compromise of confidentiality, access to the government clinics and “not remembering the date for a follow‐up visit.” There was overall excitement about and willingness to use CAB‐LA across all the groups of sex workers (female, male and transwomen). However, the willingness to use CAB‐LA was more among the FSWs who have taken oral PrEP earlier and realized its benefits as well as those who have taken Depo‐Provera contraceptive injection.	[74]
	Poteat, T. et al. (2021)	Africa	Observational study	36	Injectable PrEP (hypothetical products)	Dislike of daily dosing was a common theme and the most common reason for preferring injectable or topical over oral formulations. Trans women mentioned a reduction in health facility visits as an advantage of injectable PrEP over oral PrEP, as reports of discrimination by healthcare providers were ubiquitous.	[75]
	Tagliaferri Rael, C. et al. (2021)	North America	Observational study	15	Injectable PrEP (hypothetical products)	With respect to self‐injections, many participants liked that: (1) the process was similar to that used to self‐injected hormones; (2) transgender women could inject themselves without relying on others; (3) self‐injection eliminated a routine doctor's visit; and (4) injections could be administered in the comfort of one's own home. In their narratives, however, participants recognized that self‐injections could carry a higher margin of error. With respect to injections during drop‐in hours, many transgender women liked: (1) the convenience of short wait/visit times and the flexibility of not needing an appointment, and (2) that it felt like for some people, injections given by providers were safer. On the other hand, participants disliked that they might see others from the community during drop‐in hour.	[76]
**Care providers**							
	Ngongo, P. B. et al. (2017)	Africa	Observational study	165	Injectable PrEP (hypothetical products)	There was a difference of opinion on mode of administration, with healthcare providers and MSM preferring oral PrEP and other groups (FSW, youth) opting for injectable PrEP.	[34]
	Calder, B. J. et al. (2018)	North America	Observational study	21	Injectable PrEP (hypothetical products)	Medical practitioners recognized that injections and implants could ensure better adherence but were sceptical that achieving better adherence would motivate MSM to choose them over daily oral PrEP.	[37]
	Kerrigan, D. et al. (2018)	North America	Observational study	26	CAB‐LA (enacted preference)	Some providers may be more cautious in prescribing injectable versus oral PrEP as it is harder to clinically manage and more difficult to discontinue quickly.	[40]
	Hershow, R. B. et al. (2019)	North America	Observational study	20	Injectable PrEP (hypothetical products)	Several providers felt that injectable PrEP would be preferred since PWID would have one less thing to worry about each day and would find adhering to a daily pill difficult. Some providers mentioned retention issues when asked whether they thought PWID would prefer oral or injectable PrEP, stating PWID would have difficulty with injectable PrEP due to the need for regular visits to a provider. Some providers expressed concerns around PWID developing resistance mutations if clients initiated injectable PrEP and were not regimented about attending follow‐up visits.	[77]
	Xavier Hall, C. D. et al. (2021)	North America	Observational study	11	Injectable PrEP (hypothetical products)	Injectables would lessen daily cognitive burden on users; however, elements of the regimen were seen as increasing complexity and reducing trialability. All providers suggested that the dosing schedule and frequency of visits could be barriers. A subset of providers favoured seeing patients more frequently to monitor progress.	[78]
**PWID**							
	Biello, K. B et al. (2019)	North America	Observational study	33	Injectable PrEP (hypothetical products)	Most participants were enthusiastic about injectable PrEP and expressed greater interest in injectables than daily oral PrEP. A primary reason for preferring injectable PrEP (over oral PrEP) was easier adherence. For homeless or marginally housed PWID, participants discussed the benefits of not needing to carry or store medication. Most participants perceived that getting an injection every 2 months would not be a major barrier, but some worried about attending appointments every 2 months for injections. They suggested adherence strategies, like appointment reminder calls and distributing injectable PrEP at local pharmacies. Some participants felt they would need more information about injectable PrEP: who might give the injections, side effects and costs compared to oral PrEP. Some concerns were related to general mistrust of medical systems/providers and apprehension about being injected with unknown substances. Some worried that injectable PrEP could affect their high.	[79]
	Footer, K. H. A. et al. (2019)	North America	Observational study	31	Injectable PrEP (hypothetical products)	Participants raised concerns that they would have less control over side effects with injectable PrEP. In the context of discussing irregular visits to a primary healthcare provider, sex workers and PWID expressed a preference for a longer lasting injectable (6 vs. 3 months) and implantable (12 months) PrEP. Some PWID had concerns about forgetting to go back for renewed protection. Long‐lasting delivery methods removed the concerns about daily adherence.	[80]
	Allen, S. et al. (2020)	North America	Observational study	48	Injectable PrEP (hypothetical products)	Participants explained that the availability of a long‐acting PrEP option, whether in pill or injectable form, would alleviate many of the barriers PWID face in taking PrEP.	[81]
	Shrestha, R. et al. (2020)	North America	Observational study	234	Injectable PrEP (hypothetical products)	67.1% and 25.6% of participants reported having heard of oral PrEP and injectable PrEP, respectively. The most frequently reported concern about injectable PrEP was the potential for long‐term side effects (76.9%). Participants reported slightly less concern about the possibility of waning efficacy of injectable PrEP (37.6%) or incomplete protection against HIV (33.3%), followed by the cost of injectable PrEP (27.4%), fear or dislike of needles (24.8%) and having to return to the clinic for injection of injectable PrEP every 2 months (20.5%). 73.5% reported that they would be willing to use injectable PrEP. Factors independently correlated with willingness to use injectable PrEP included female sex (aOR = 2.181, *p* = 0.018), being engaged in healthcare (aOR = 2.919, *p* = 0.023), having high perceived risk for HIV transmission (aOR = 3.255, *p* = 0.007) and having ever used oral PrEP previously (aOR = 3.284, *p* = 0.017).	[82]
	International Network of People Who Inject Drug (2022)	Not available	Observational study	Not available	Injectable PrEP (hypothetical products)	Several participants highlighted that although daily oral PrEP is already available through community‐led organizations, other PrEP modalities, such as injectables (CAB‐LA), are either not available to PWID or people are unaware of its availability or how to access it. This is despite preferences for such modalities due to greater perceived efficacy, tolerability and convenience when compared to daily oral PrEP	[83]
**Sex workers**							
	Mack, N. et al. (2014)	Africa	Observational study	133	Injectable PrEP (hypothetical products)	In Kenya, the majority of FSW preferred an injectable over other formulations because one dose would last for a prolonged period and require little user intervention. Injections were perceived as relatively private. A few women described alcohol use as potentially interfering with their ability to take daily pill but not posing a problem with injections.	[64]
	Quaife, M. et al. (2018)	Africa	Observational study	609	Injectable PrEP (hypothetical products)	Adult women and FSWs significantly disliked oral PrEP and favoured injectable products. Neither adult women nor adolescent girls found the vaginal ring appealing but an injectable product was favoured by all groups.	[57]
	Minnis, A. M. et al. (2020)	Africa	Observational study	807	Injectable PrEP (hypothetical products)	MSW expressed greater preference for two injections compared with implants (*p* < = 0.009). MSW disliked injection on the buttocks (*p* = 0.01).	[48]
	Ashodaya Samithi (2021)	South Asia	Observational study	165	CAB‐LA (hypothetical products)	Most community members were excited about CAB‐LA, particularly male sex workers and trans women sex workers. However, there were queries about CAB‐LA side effects, HIV resistance, adherence support, and so on. Trans women sex workers expressed that CAB‐LA would be a good option if it does not interfere with gender‐affirming hormones. Some expressed worry about the pain on the injection site. Though many MSW and trans women sex workers were interested in CAB‐LA, some were sceptical and would prefer to wait and see its use in India before they start taking it for themselves. Many FSWs relate CAB‐LA with Depo‐Provera injection. The long‐acting prevention tool would relieve them from the worry of getting HIV and would ensure the daily oral tablet is not required. The primary concern regarding taking the injection from government hospitals remained long waiting times, compromise of confidentiality, access to the government clinics and “not remembering the date for a follow‐up visit.” There was overall excitement about and willingness to use CAB‐LA across all the groups of sex workers (female, male and transwomen). However, the willingness to use CAB‐LA was more among the FSW who have taken oral PrEP earlier and realized its benefits as well as those who have taken Depo‐Provera contraceptive injection.	[74]
	Footer, K. H. A. et al. (2019)	North America	Observational study	31	Injectable PrEP (hypothetical products)	Participants raised concerns that they would have less control over side effects with injectable PrEP. In the context of discussing irregular visits to a primary healthcare provider, sex workers and PWID expressed a preference for a longer lasting injectable (6 vs. 3 months) and implantable (12 months) PrEP. Some PWID had concerns about forgetting to go back for renewed protection. Long‐lasting delivery methods removed the concerns about daily adherence.	[80]
**Other populations**
Adults reside in high‐burden districts	Govender, E. et al. (2018)	Africa	Observational study	112	Injectable PrEP (hypothetical products)	Most women favoured injectable PrEP as it offered a longer period of protection against HIV, was easily adopted into existing family planning routines and did not require daily administration. Many wanted a product that was safer, less user‐reliant and required minimal negotiation/partner support. Product sensitivity with injectables and the intravaginal ring were raised by many men who were concerned about side effects and allergic reactions. Women were concerned that the injectable could lead to weight gain, causing them to not feel sexy.	[84]
Heterosexual men	Cheng, ChihYuan et al. (2019)	Africa	Observational study	202	Injectable PrEP (hypothetical products)	Forty‐eight percent (*n* = 85) of participants chose injectable PrEP, with 33% (*n* = 58) and 20% (*n* = 25) choosing oral PREP and condoms, respectively. Men with children had a 22% higher likelihood of choosing injectable PrEP relative to childless men. Men who have ever had unprotected anal sex had a decreased probability of 42% of choosing injectable PrEP compared to those who never had unprotected anal sex. An increase of 1 point in the self‐rated risk attitude score was associated with a 3% increased likelihood of choosing injectable PrEP.	[85]
Adults reside in fishing communities	Kuteesa, M. O. et al. (2019)	Africa	Observational study	805	Injectable PrEP (hypothetical products)	Among women, there were comparable levels of demand for oral PrEP, injectables and implantable products; however, the uptake of an intravaginal ring was predicted to be substantively lower. Class 2 women (more likely to be younger and without prior intimate partner violence) had strong, positive preferences for the longer‐lasting products compared to oral PrEP, and significantly valued secrecy of use.	[86]
HIV‐uninfected healthy at‐risk adults	Laher, F. et al. (2020)	Africa	Observational study	38	Injectable PrEP (hypothetical products)	Many participants voiced a need for an HIV prevention method requiring infrequent administration (lasting at least a month). Reasons cited included: forgetfulness when using daily methods; daily methods interfered with lifestyle priorities; costliness of transportation for methods that required frequent clinic visits; and product inaccessibility due to of clinic operating hours.	[87]
						2. Injections were favoured by male and female participants and were perceived as advantageous because they were thought to work quickly systemically. There was general support for injectable PrEP and vaccine innovations. Although injections were perceived as painful, participants were willing to use them because they were a route of administration linked with efficacy, discretion and long‐lasting protection.	
Healthy adults at low HIV risk	Tolley, E. E. et al. (2020)	North America, Africa, Latin American and Caribbean	Randomized control trial	199	CAB‐LA (enacted preference)	At baseline, participants most liked the idea that a PrEP injectable would be easier to use than other methods, might protect against HIV and could provide a longer duration of protection than other methods. However, one‐third expressed concerns about potential side effects and pain. After receiving a first injection at week 6, at least 50% of participants in cohort 1 and approximately 75% or more of participants in cohort 2 rated the number, frequency, location and duration of injection as highly acceptable. More non‐U.S. participants reported pain to be unacceptable than did U.S. participants at both timepoints. Participants receiving CAB‐LA injections were more likely to find the location of the injectable—in the buttocks—acceptable than those receiving saline injections. After their first injection, approximately 40% of participants in the CAB‐LA arm and 75% of cohort 1 placebo participants reported either experiencing no pain or pain that was highly acceptable. After each injection visit, participants in the placebo arm reported significantly higher acceptability of physical experiences than those in the CAB‐LA arm. Over the injection phase of the trial, 27 participants permanently discontinued injectable product use. Stated reasons for discontinuation included product‐related side effects, inability or unwillingness to follow study procedures, abnormal lab values, reactive HIV tests and desire for pregnancy.	[88]
						6. In univariate models, future interest in use was positively associated with non‐U.S. versus U.S. region (OR 2.9, *p* = 0.0002), with higher levels of acceptability for product attributes (OR 4.77, *p* < 0.0001) and for physical experiences (OR 1.6, *p* = 0.0002), having higher levels of altruism (OR 1.96, *p* < 0.0001) and fewer total injection site reactions (OR 0.9, *p* = 0.004). Among participants born female, having ever used a contraceptive injectable was associated with a significant increase in future interest in use (OR 3.4, *p* = 0.001). 7. In multivariable models, future interest in use was strongly associated with the composite acceptability score for product attributes (OR 4.84, *p* < 0.0001). Non‐U.S. participants (OR 2.9, *p* = 0.0003) and those with higher baseline levels of altruism (OR 1.52, *p* = 0.005) had higher future interest in use.	

Abbreviations: aOR, adjusted odd ratios; CAB‐LA, long‐acting injectable cabotegravir; CI, confidence interval; DCE, discrete choice experiment; FDA, Food and Drug Administration; FIIP, future interests in injectable PrEP; FSW, female sex worker; HIV, human immunodeficiency virus; MPTs, multipurpose prevention technologies; MSM, men who have sex with men; MSW, male sex worker; PrEP, pre‐exposure prophylaxis; PWID, persons who inject drugs; WWID, women who inject drugs.

### 3.2 Quality assessment

We assessed 53 full‐text articles for risk of bias using JBI criteria. Overall, included studies met most checklist criteria, indicating a low‐to‐medium risk of bias for individual studies. There were specific limitations by the study design/checklist. For example, across 27 qualitative studies, all lacked a statement locating the researcher culturally or theoretically. Only one mentioned the influence of the researcher on the research and vice versa. We applied the JBI checklist for cross‐sectional studies to 22 references with quantitative outcomes. All used appropriate statistical analysis; however, five did not identify confounding factors or state strategies to address them. Four studies were assessed with the checklist for randomized controlled trials, but none met all criteria. The main limitation was a lack of clarity as to whether the outcome assessors were blinded to treatment assignments and if participants were analysed in the groups to which they were randomized.

### 3.3 CAB‐LA‐specific findings

Six studies examined values and preferences for CAB‐LA: three from the ECLAIR trial [40, 41, 43]; one each from HPTN 077 [88] and HPTN 084 [63]; and a qualitative study with sex workers (men, women, trans women) in India [74]. Across ECLAIR, a U.S.‐based placebo‐controlled trial among men at low risk for acquiring HIV, participants reported willingness to use or high satisfaction with CAB‐LA [40, 41, 43]. Injection pain was common [41], yet most reported satisfaction with the product despite side effects and pain/discomfort [43]. HPTN 084 participants overwhelmingly preferred CAB‐LA to daily oral PrEP [63]. Most HPTN 077 participants found the number, frequency and location of injections initially acceptable [80], though future interest in CAB‐LA was higher in non‐U.S. sites. HPTN 077 participants in the placebo arm reported higher acceptability of physical experiences [88]; however, there was no relationship between injection site pain and future interest in CAB‐LA [88]. ECLAIR participants and SSA women from HPTN 084 appreciated that CAB‐LA afforded more privacy and improved adherence over pills [16, 63]. Finally, sex workers in India were willing to use CAB‐LA, especially those with prior oral PrEP or depot‐medroxyprogesterone acetate (DMPA) experience [74].

### 3.4 Affective attitude

Nearly, all studies reported on affective attitude (Table 5). Most explored stated preference (i.e. preference for hypothetical products or attributes). Less common were reports of enacted preference (i.e. experimentation with multiple modalities and subsequent choice of a preferred product) or preference based on experience with placebos or active injectables. Where possible, we provide details to clarify enacted preference or preference based on experience.

**Table 5:** Overview of articles by acceptability constructs and groups

	MSM	Women	Trans men and women	Adolescents and young people	PWID	Care providers	Sex workers (male and female)
**Affective attitude**	[28, 31–45, 47–50, 52, 69, 85]	[34, 48, 55, 57–61, 63, 64, 74, 84, 86, 88]		[34, 51, 64, 66, 68]	[61, 77, 79‐83]	[34]	
**Burden—ease of use and facilitator**							
Ease of use and convenience	[33, 37, 46, 71]	[62, 87]				[78]	
Injections circumvent daily pill‐taking burden	[31, 53, 69]		[72, 75]		[79, 80]		
Fit with family planning		[61, 64, 74, 84]					[74]
Frequency of injection	[32, 44]	[48]	[76]	[51]			
Trained, sensitive providers	[44]	[55]					
**Burden—concerns and challenges**							
Dislike/fear of needles and pain	[33, 37, 39, 41, 44, 46]	[59, 61, 63, 65]			[61, 79]		[55, 64, 74]
Side effects	[32, 33, 36, 39, 40]	[62]			[79, 80, 82]		
Invasiveness and body location of the injection	[40, 41, 48]	[55]		[48, 64]			
Logistical challenges	[36, 46, 69, 73]	[62]	[36, 46, 73]	[51]			
Lack of control	[37, 69]	[55]					
**Ethicality**	[29, 31, 69]	[61, 62, 64, 65, 84, 86]	[72, 73, 75, 76]	[51]	[61, 79]		[64]
**Intervention coherence**				[67]			
**Opportunity costs**		[84]	[74, 82]		[79]		[74]
**Perceived effectiveness**	[36, 39, 40, 69]	[62]		[65]	[82]	[77]	
**Self‐efficacy**				[68]	[79, 80]		[74]

Abbreviations: PWID, persons who inject drugs.

#### 3.4.1 U.S.‐based MSM

Injectable PrEP was often, though inconsistently, preferred among U.S.‐based MSM. ECLAIR participants reported higher satisfaction with CAB‐LA over oral PrEP and higher interest in future use [40, 43]. Although 25% of participants receiving CAB‐LA were dissatisfied with the pain/discomfort associated with the injection, 74% were satisfied enough to continue CAB‐LA [43]. Other studies also reported a willingness to receive injectable PrEP [28] or a preference for injectable PrEP compared to daily oral PrEP [32, 39, 44, 46], on‐demand PrEP [35, 52], penile or rectal gels or anal suppositories [44, 52]. However, two studies reported a preference for subdermal implants over injectables [33, 46]. Others reported a preference for daily oral PrEP over injectables [33], since adherence to daily oral PrEP was already high [37]. Overall, there were conflicting reports about how oral PrEP experience influenced preferences. Two studies reported greater interest in potentially using injectable PrEP among oral PrEP‐naïve versus oral PrEP‐experienced participants [36, 50], but a third study reported daily PrEP users as more likely to endorse future injectable PrEP use [52]. Some MSM noted the value of long‐acting PrEP could increase if proven more effective or lower cost than oral PrEP [37]. Three studies noted a preference for the most effective method [32, 33, 36].

U.S.‐based adolescents assigned male at birth more often preferred condoms or yearly implants compared with injectables or quarterly implants [51]. More Black and Latino MSM youth preferred daily oral PrEP, followed by PrEP implants, then injectables [66].

#### 3.4.2 MSM in other regions

Globally, preferences for injectable PrEP among MSM varied. MSM in India preferred injectables administered monthly or every 2 months over daily oral PrEP [29]. In China, most were willing to use injectable PrEP over pills [42] or willing to use on‐demand PrEP, followed by injectable PrEP, then daily oral pills [47]. Meanwhile, MSM reported a preference for rectal microbicide gels over injectables in Vietnam [31], oral PrEP over injectables in Kenya [34], injectables over implants in South Africa [48] and injectables followed by daily PrEP, then on‐demand PrEP in Latin America [49]. Ukrainian choice‐based analyses reported a stated preference for injectable PrEP for younger [45], well‐educated MSM [38, 45] and those living off a lower income [38], with cost an important consideration.

#### 3.4.3 Trans men and women

Values and preferences research among trans men and women was typically grouped with MSM, making it challenging to disentangle findings for the trans community. Of the disaggregated research on affective attitudes, trans women in SSA reported preferring injectables to oral or topical formulations [75], trans women sex workers in India reported excitement for CAB‐LA [74], and trans men and women from various geographies in a multi‐country report often referred to injectables as the preferred PrEP modality [73].

#### 3.4.4 Cisgender adult women and adolescent girls

Feelings towards injectable PrEP also varied among heterosexual, cisgender women. In the U.S., there was a slight preference for pills over injections in a study presenting different PrEP modalities [13] but a preference for injectable PrEP over daily pills in others [60, 61]. Some preferred injectables given familiarity with injections for other medications/drugs [60].

Interest in injectable PrEP also varied in SSA, though several studies highlighted a strong preference for injectables. In a qualitative sub‐study assessing preferences for HIV prevention formulations among participants in a phase III trial of DVR, the ring was most preferred, followed by implants, injectables, male condoms, then oral pills [55]. In a discrete choice experiment, Ugandan women reported comparable levels of demand for oral PrEP, injectables and implants [86]. However, in several SSA studies, women reported preferences for injectable or implantable formulations over daily oral PrEP [34, 48, 54, 57, 64, 84]. Women in HPTN 084 reported an overwhelming preference for CAB‐LA compared to daily oral PrEP [63]. African women in HPTN 076 (assessing rilpivirine injections) more strongly endorsed injectable PrEP than U.S.‐based participants, and even more so if the injectable offered HIV and pregnancy prevention together [59].

In the TRIO study, which compared women's modality preferences for placebo multi‐purpose prevention technologies (MPTs) in South Africa and Kenya, injections were the most popular modality (over oral pills or vaginal rings) and achieved the highest adherence [58]. However, more participants stated they would choose an MPT ring or pill over an injection that solely prevented HIV [58]. Sex workers in India had a higher willingness to use CAB‐LA if they had previous exposure to oral PrEP or DMPA [74]. Finally, adolescent girls and young women in SSA [34, 64, 68] reported a preference for injections over daily pills. In a study of MPTs in SSA, participants rated the acceptability of injectables significantly higher than for rings or pills [89]. Malawian adolescents were particularly interested in facility‐based injections [68].

#### 3.4.5 PWID

Compared to some groups, values and preferences research is lacking for persons who inject drugs (PWID). Among studies examining U.S.‐based PWID, there was greater interest in injectables [79, 82] or implants [80] compared with daily oral PrEP, given irregular visits to primary health centres. PWID [81] and providers [77] reported that the availability of injectable PrEP could alleviate barriers to PrEP adherence for PWID. Some indicated that other PWID would be willing to try injectable PrEP given familiarity with needles [61, 79]. Despite low awareness of injectable PrEP, PWID from a multi‐regional assessment reported a preference for injectable PrEP over daily oral PrEP due to greater perceived efficacy, tolerability and convenience [83].

### 3.5 Burden—ease of use and facilitators

Following affective attitude, burden was the most commonly reported TFA construct (reported in 34 references), including ease of use, facilitators or challenges.

#### 3.5.1 Ease of use and convenience

ECLAIR participants reported relative ease of use of injectables compared to oral PrEP, which reduced long‐term adherence concerns [40]. Among MSM, injectable PrEP was considered an easy‐to‐use, convenient option compared to daily pills [33, 46] that provided privacy and adequate duration of protection [33]. Some MSM noted that injectables presented a therapeutic simplification over oral PrEP that suited busy lifestyles [37, 71]. U.S.‐based women also reported injectable PrEP as convenient [62]. South African women in a vaccine efficacy trial reported that injectable PrEP fulfilled the need for an effective, discreet method requiring infrequent administration [87].

#### 3.5.2 Injections circumvent daily pill‐taking burden

A major benefit of injectables was the reduced burden of daily pill‐taking. Current PrEP users were more likely to see not forgetting doses as an advantage of injectables compared to people living with HIV (PLWH) [70]. ECLAIR participants and women in MTN‐003D (assessed preferences for oral PrEP, gel and other formulations [54]) reported the relative ease‐of‐use of injectables and implantables reduced end‐users’ fears about maintaining long‐term adherence. Women in HPTN 084 said injections circumvented fears of forgetting to take pills, which could happen due to lifestyle considerations, such as late‐night work, travel or drinking alcohol [63]. Similarly, PWID reported that injectable PrEP reduced concerns about daily dosing [80] and more easily fit into clinical care schedules [79].

MSM in Vietnam [31, 53] and U.S.‐based Black and Latino youth [66] and MSM [69] reported injectable PrEP as beneficial to reducing the anxiety of remembering to take a daily pill. Trans women in South Africa specifically noted that dislike of daily dosing was a common reason for preferring injectable formulations [75], while U.S.‐based trans women appreciated not having to adhere to a daily product to have reliable protection [72]. Some U.S.‐based providers said injections could reduce the burden of remembering to take daily pills for users, yet dosing schedules and frequency of visits were cautioned as adherence barriers [78].

#### 3.5.3 Fit with family planning

In the U.S. and South Africa, adult [13, 61, 84] and young women [64] reported that injectables fit into existing family planning routines and modalities for some (e.g. receiving contraceptive injectables in the buttocks), which was an important facilitator of injectable PrEP use. Some female sex workers (FSWs) in India compared CAB‐LA with taking DMPA [74].

#### 3.5.4 Frequency of injection

Frequency of injection was an important attribute, including among U.S.‐based MSM who found a 3‐month (or longer) injectable preferable to shorter durations [32, 44]. Some South African women preferred receiving bimonthly injectable PrEP at a health facility but would consider a pharmacy if dosed less frequently [48]. For U.S.‐based sexual and gender minority adolescents, ease of use would be supported by administration in the arm, reducing the number of annual injections, and—though not yet recommended—self‐administration similar to gender‐affirming hormones [51]. Many U.S.‐based trans women also favoured self‐injection, though acknowledged this could yield errors [76].

#### 3.5.5 Trained, sensitive providers

Some ECLAIR participants felt that friendly nurses trained to give intramuscular injections would improve injectable PrEP use [41]. Per U.S.‐based MSM, attitudes or behaviours of personnel administering the injection could affect preferences for this modality [44]. Some women in SSA expressed concerns about the qualifications of clinical staff to perform “invasive procedures” like injections [55].

### 3.6 Burden—concerns and challenges

#### 3.6.1 Dislike/fear of needles and pain

Dislike or fear of needles was mentioned by FSWs and other women in SSA and India [55, 64, 74], adolescents in Malawi [68] and South Africa [64], U.S.‐based MSM [33, 41, 44, 46], and former injection drug users and other women in the U.S. [61, 63, 79]. Some former PWID found the use of needles “triggering,” as they were reminders of their history of drug use. However, dislike/fear of needles was somewhat countered by the high level of protection provided by injectable PrEP [44]. Some experienced PrEP users also reported fear of poorly administered injections [71]. Several young women in an MPT trial in South Africa [89] and many women in the rilpivirine trial [59] noted fear of pain upon initially seeing the needle, though this typically subsided after experiencing the injection. Few rilpivirine trial participants reported pain as unacceptable [59]. South African women also acknowledged that pain is temporary following injection, and soreness subsides [65].

#### 3.6.2 Side effects

Concerns over side effects, including not having control over side effects, were mentioned by PWID [79, 80], adolescents [51] and MSM [32, 36, 39]. Some U.S.‐based women reported that side effects, including potential interference with pregnancy, were barriers to uptake [62]. Despite concerns over side effects, ECLAIR participants noted a willingness to deal with CAB‐LA side effects if found to be effective [40]. Beyond side effects, MSM [32, 39] and PWID [82] in the U.S. expressed concerns over the long‐term effects of injectable PrEP.

#### 3.6.3 Invasiveness and body location of the injection

Invasiveness of injection location (i.e. buttocks) was a barrier for women shown hypothetical prevention modalities at the end of a DVR trial in SSA [55]. Adolescent girls in South Africa were uncomfortable with having to remove their skirts for the injection, instead preferring an injection in the arm [48, 64]. Several ECLAIR participants also noted embarrassment from needing to expose one's buttocks [40, 41], and some MSM and heterosexual men in South Africa had concerns about the buttocks as an injection site [48].

#### 3.6.4 Logistical challenges

MSM and trans people expressed logistical concerns, including needing to regularly return for appointments [36, 46, 73]. By contrast, regular visits were not considered a barrier by some U.S.‐based MSM currently using oral PrEP since it also required regular clinical visits [69]. Logistics were a concern for men in a PrEP demonstration project inconvenienced by scheduling follow‐up appointments [69]. U.S.‐based women were concerned with appointment frequency [62], and adolescents disliked how injectable PrEP has a short duration/coverage, requiring frequent appointments [51].

#### 3.6.5 Lack of control

MSM mentioned lack of control over their bodies and medication administration, particularly compared to pills that are fully under user control, as a disadvantage of injectables [37, 69]. Lack of control worried PrEP‐experienced users [71], who were more likely to see the loss of control or adverse effects as barriers to injectables than PLWH [70]. In a PrEP demonstration project, men liked the control of taking a daily pill over using an injectable [69]. Women in SSA expressed concern about injectable reversibility, that is the ability to remove or discontinue product use if there are concerns about safety or side effects [55].

### 3.7 Ethicality

Sixteen studies reported on ethicality [29, 31, 40, 51, 54, 61–65, 69, 79, 84, 86, 89, 90]. One of the most desirable aspects of injectable PrEP was its ability to be concealed. Women in the U.S. [62], FSWs in South Africa [64, 78] and young women using placebo MPTs in SSA [89] described injectables as offering discretion and confidentiality. Participants in CAB‐LA studies also noted a preference for injectables by those valuing confidentiality, as injectables afford more privacy than pills [40, 63]. SSA women reported that longer‐lasting products were more acceptable to women valuing secrecy [86], discretion and invisibility [65] as well as women needing maximum protection and minimal partner negotiation [84]. Facility‐based administration was appealing for women hiding pills at home from partners, family or children [54]. MSM in several studies also reported that injectable PrEP obviates the need to conceal PrEP at home or carry pills while travelling [29, 31, 69]. Adolescents assigned male at birth also liked that injectables could be easily concealed and included as part of routine check‐ups [51].

Injectable PrEP was typically considered a better fit for people comfortable with needles [40, 61, 79], though some studies challenged this assertion. Some PWID noted social or cognitive burdens in that some with a history of injection drug use, especially those in recovery, could be triggered by injections [61, 79]. Other PWID worried that injectable PrEP could “affect their high” [79]. Women with a history of medication‐related injections (e.g. insulin pumps, steroid injections, etc.) expressed reticence to add another injectable to their regimens [61].

Finally, injectables may be appropriate for the unhoused [36], those engaging in more frequent sex or those looking to reduce perceived stigma related to oral PrEP [71]. Some oral PrEP users reported that, because injectables offered discretion, they could reduce social stigma [71]. Additionally, some trans people felt that injectable PrEP reduced the risk of carrying drugs that might expose them as being vulnerable to HIV or being falsely assumed as HIV positive [73]. Nevertheless, stigma was also a barrier for trans women attending drop‐in hours at a health facility for injectable PrEP [76]. Trans women in South Africa said reducing the number of healthcare visits for injectables could mitigate discrimination from healthcare providers [75]. Some trans women reported feeling comfortable with the idea of self‐administering at home, much like gender‐affirming hormones [72].

### 3.8 Intervention coherence

One study addressed intervention coherence. U.S.‐based LGBTQ youth questioned how injectable PrEP would protect them, and in some cases, made incorrect assumptions about how biomedical prevention works [67]. Participants requested information about the rationale for the injection site and whether injectables would interfere with other recreational, over‐the‐counter or gender‐affirming drugs [67].

### 3.9 Opportunity costs

Six studies reported on opportunity costs [38, 45, 72, 74, 79, 84]. Trans women in the U.S. and India worried that injectables could interfere with gender‐affirming hormones [72, 74], with some feeling more research was necessary to explore these interactions [72]. U.S.‐based trans women also discussed the potential effect of injections in gluteal muscles due to silicone implants and concerns about scarring [72]. South African women reported concerns about weight gain and poor body image as a potential result of injectable PrEP [84]. U.S.‐based PWID raised concerns over the monetary costs of injectables relative to oral PrEP [79], and European MSM [38, 45] noted cost as a major influencer when considering different hypothetical products, ranking cost as more important than dosing frequency.

### 3.10 Perceived effectiveness

Nine studies addressed perceived effectiveness [36, 39, 40, 62, 65, 69, 71, 77, 82]. Some MSM were interested in CAB‐LA [40] or injectable PrEP more generally [36] because it was perceived as an effective modality, particularly in instances of unexpected HIV risk or condomless sex. Some U.S.‐based women perceived injections to be more effective than pills [62], and some South African youth perceived that injections provided greater protection than vaginal rings since the injection is systemic and readily absorbed [65]. However, others expressed concerns over the experimental nature of long‐acting anti‐retroviral therapy (ART) and PrEP, wanting more scientific evidence [71] or FDA approval [69] before using. MSM were concerned about diminishing, waning or incomplete protection associated with injectable PrEP [36, 39, 69]. Among men in a U.S.‐based PrEP demonstration project, the biggest barrier to switching to injectables included concerns about safety, efficacy and not trusting protection from a single shot [69]. Some providers of PWID were concerned over ARV resistance if participants did not return for follow‐up appointments [77]. However, PWID were less concerned about incomplete protection than longer‐term health effects [82].

### 3.11 Self‐efficacy

Five studies addressed self‐efficacy [68, 74, 77, 79, 80], which mapped to some logistical concerns noted under *Burden* given the focus on participant's assessment of their ability to adhere to injectable PrEP. There was concern among PWID [79, 80] and their providers [77] over PWID's ability to consistently attend appointments. Participants countered concerns by discussing adherence strategies [76, 79]. Worry over forgetting appointments was also reported by some Malawian adolescents [68] and sex workers in India, who were challenged by long wait times at government hospitals and not remembering appointment dates [74].

## 4 DISCUSSION

Since 2010, a wealth of literature has explored values, preferences and acceptability related to injectable PrEP. This review found an overall preference for and much interest in injectable PrEP, including CAB‐LA. However, there was variation in preferences within and across groups and geographies. This is consistent with evidence that preference for injectable contraceptives also varies by location, with women in low‐income countries more likely to prefer an injectable than women in high‐income countries [91]. The variation also highlights the importance of including injectable PrEP in a menu of prevention options, allowing end‐users to interface with providers to choose a PrEP modality that suits their lifestyle and supports effective use. Moreover, as this review included a variety of groups and regions, it is important to consider how individual risk perceptions are associated with interest in product use [92, 93] and acceptance [94], as has been demonstrated for oral PrEP use.

Most studies were conducted before CAB‐LA efficacy data were published and CAB‐LA had received regulatory approval, therefore, among participants who had not used CAB‐LA. Participants were typically asked about preferences for hypothetical injectables compared with other PrEP products. More recent efficacy trials have tested placebo and active injectables, including CAB‐LA, rilpivirine and lenacapavir (as a semi‐annual subcutaneous injectable [95]). Evidence now suggests that CAB‐LA is efficacious and safe for sexual exposure [96], thus receiving regulatory approval in the United States, Australia, Zimbabwe, South Africa and Malawi, which may overcome some concerns expressed within included studies. There is also increasing information on issues such as safety during pregnancy, although data remain limited.

When considering the rollout and scale‐up of injectable PrEP, policy makers and healthcare providers should consider lessons learned from the slow and inequitable uptake of oral PrEP [1]. Assessing differentiated service delivery models, addressing provider bias and creating patient‐centred decision tools may enable more equitable implementation of injectable PrEP [97], in addition to conducting research on how end‐users make real‐time decisions. Implementation plans are underway to offer CAB‐LA alongside other PrEP options [98], providing opportunities to assess how people choose, continue and switch between products. It is important to offer a choice of products to potential end‐users and that healthcare providers understand and respond to end‐users’ concerns, as identified here, to help make informed choices. As CAB‐LA becomes available, future research might also explore end‐user costs in accessing regular injections, including transportation fees to appointments, as cost was identified as a potential barrier to oral PrEP use [99].

Preferences for different modalities is driven, in part, by acceptability constructs. This is consistent with research indicating that preferences for prevention modalities depends on individual characteristics and may change over time [100]. Beyond affective attitude, for which there was no consistent pattern across groups and geographies, burden was most commonly described. Several concerns were raised, including fear of needles and/or injection site pain, side effects, invasiveness of injection location, logistical challenges and lack of control or reversibility. Nevertheless, facilitators of injectable PrEP included longer‐lasting coverage and peace of mind. Preference for injectable PrEP may also be driven by age or ethicality, with different issues influencing acceptability across groups. Injectable PrEP may be beneficial for those seeking discretion or having challenges negotiating product use with sexual partners. It may fit within the lifestyles of those experienced with taking other medicine by injection (e.g. contraceptives or gender‐affirming hormones), though unhappy associations with needle use may be concerning for PWID in recovery. Importantly, injectable PrEP presents a strong option for those having difficulty taking or unable to take daily or event‐driven PrEP, including adolescents.

### 4.1 Limitations

Strengths of this review include being the first to comprehensively assess acceptability for injectable PrEP. We included high‐quality literature, as indicated by a low overall risk of bias, with findings organized by a theoretical framework identifying the most salient constructs related to acceptability. This review also presents insights for various geographies and groups, including under‐researched groups like trans men and women. Although we used a systematic approach that reduced different sources of biases, some may still be present (e.g. publication bias). A key limitation is that most studies reported a preference for hypothetical products rather than enacted preference. The research was conducted prior to FDA approvals and the WHO recommendation, which affected end‐user perceptions. Values and preferences may shift as end‐users can use and choose between approved products.

Currently, CAB‐LA is recommended to prevent HIV via sexual exposure. Animal models suggest efficacy to prevent parenteral exposure [101], but research is limited, and CAB‐LA has not been specifically studied in PWID. Although PWID often have overlapping risk, this may influence their preferences. Another limitation is that certain groups or geographies are over‐represented, while others are under‐represented. We attempted to address this by stating the group/region reporting a preference, though findings suggest limited generalizability for certain groups. A limitation of CAB‐LA studies was that none presented information on injection site discreetness or lack thereof (e.g. if the injection left a visible lump or scar). Lastly, this review contained studies with different sampling methods. The quality assessments noted limitations of varying approaches, though selection bias inherent in certain designs may have captured more favourable opinions of injectables. Yet, we feel this presents a low risk to the review's interpretations given the variation in preferences reported across populations and geographies.

## 5 CONCLUSIONS

This review found an overall preference for and interest in injectable PrEP, including CAB‐LA, across groups and regions. Variation reinforces the inclusion of injectable PrEP as one of many prevention options offered to end‐users, whose preferences and needs may shift over time. Injectable PrEP presents an opportunity to address adherence‐related challenges associated with oral PrEP and may be a better lifestyle fit for individuals seeking discretion or are familiar with needles. However, end‐users reported concerns related to fear/pain, logistical challenges and waning levels of protection. More research is necessary to explore enacted preference for end‐users exposed to ARV‐containing injectables.

## COMPETING INTERESTS

The authors declare no competing interests.

## AUTHORS’ CONTRIBUTIONS

The study was conceptualized by RB, MR and RS. LL and ND drafted the study protocol with input from AVDS, VF and KR. LL, ND, VF and KR conducted the screening of citations, with AVDS helping to resolve differences in eligibility determination. LL and ND abstracted and analysed data. All co‐authors contributed to data interpretation. LL drafted the initial manuscript, with all co‐authors contributing to subsequent iterations. All co‐authors have reviewed and approved the final version.

## FUNDING

This work was made possible by the generous support of the American people through the U.S. President's Emergency Plan for AIDS Relief (PEPFAR) and the U.S. Agency for International Development (USAID) cooperative agreements 7200AA19CA00002 and 7200AA21CA00011.

## DISCLAIMER

The contents are the responsibility of the EpiC and MOSAIC projects and do not necessarily reflect the views of PEPFAR, USAID or the U.S. Government. WHO staff time was supported by grants from Unitaid and the Bill & Melinda Gates Foundation, which were awarded to the World Health Organization to enable this study. The authors alone are responsible for the views expressed in this article and they do not necessarily represent the views, decisions or policies of the institutions with which they are affiliated.

## Supporting information


**Supporting Information Appendix A**: Full search terms for included databases

**Supporting Information Appendix B**: Results from rapid extraction of 38 articles briefly mentioning preferences for injectables compared with other PrEP modalities

## Data Availability

The data that support the findings of this study are available in the Supporting Information of this article.
