# Supplementary material for: Systematic review of the values and preferences regarding the use of injectable pre‐exposure prophylaxis to prevent HIV acquisition
Source: J Int AIDS Soc. 2023 Jul 13;26(Suppl 2):e26107. doi: 10.1002/jia2.26107 (PMC10805120; doi:10.1002/jia2.26107)
Supplement: Supplementary file 1 — Supporting Information Appendix A: Full search terms for included databases [file JIA2-26-e26107-s002.docx]

**Appendix A**

**Full Search Terms for Included Databases**

**PubMed**

(Cabotegravir OR "CAB-LA" OR injectable OR injection OR injections[Mesh] OR "long-acting" OR GSK1265744 OR "sustained release" OR "extended release" OR delayed-action preparations[Mesh] OR “controlled release” OR “slow release” OR “timed release” OR “prolonged-action” OR “prolonged action”) AND (“pre-exposure prophylaxis” OR “primary prevention” OR “PrEP” OR “HIV seronegativity” OR “HIV uninfected”) AND (hiv[MeSH] or "hiv infections"[MeSH] or "acquired immunodeficiency syndrome"[MeSH])

**CINAHL, Global Health**

(Cabotegravir OR “CAB-LA” OR injectable OR injection OR injections OR “long-acting” OR GSK1265744 OR “sustained release” OR “extended release” OR “controlled-release” OR “slow release” OR “timed release” OR “prolonged-action” OR “prolonged action”)

AND

(“pre-exposure prophylaxis” OR “primary prevention” OR “PrEP” OR “HIV seronegativity” OR “HIV uninfected”)

AND

(hiv OR “hiv infections” OR “acquired immunodeficiency syndrome”)

**Embase**

(Cabotegravir OR “CAB-LA” OR injectable OR injection OR injections OR “long-acting” OR GSK1265744 OR “sustained release” OR “extended release” OR “controlled-release” OR “slow release” OR “timed release” OR “prolonged-action” OR “prolonged action” OR “delayed-action” OR “delayed-action preparations”) AND (“pre-exposure prophylaxis” OR “primary prevention” OR “PrEP” OR “HIV seronegativity” OR “HIV uninfected”) AND (hiv OR “hiv infections” OR “acquired immunodeficiency syndrome”)

**Cochrane**

(Cabotegravir OR “CAB-LA” OR injectable OR injection OR injections OR “long-acting” OR GSK1265744 OR “sustained release” OR “extended release” OR “controlled-release” OR “slow release” OR “timed release” OR “prolonged-action” OR “prolonged action”) in all text AND (“pre-exposure prophylaxis” OR “primary prevention” OR “PrEP” OR “HIV seronegativity” OR “HIV uninfected”) in all text AND (hiv or “hiv infections” or “acquired immunodeficiency syndrome”) in all text

**Clinical Trials.gov**

(Cabotegravir OR “CAB-LA” OR injectable OR injection OR injections OR “long-acting” OR GSK1265744 OR “sustained release” OR “extended release” OR “controlled-release” OR “slow release” OR “timed release” OR “prolonged-action” OR “prolonged action”) | HIV prevention OR PrEP | Start date from 01/01/2010 to 10/01/2021

**ICTRP - WHO**

Title - Cabotegravir OR “CAB-LA” OR injectable OR injection OR injections OR “long-acting” OR GSK1265744 OR “sustained release” OR “extended release” OR “controlled-release” OR “slow release” OR “timed release” OR “prolonged-action” OR “prolonged action”

AND

Intervention – PrEP OR HIV prevention
