# Supplementary material for: Systematic review of the values and preferences regarding the use of injectable pre‐exposure prophylaxis to prevent HIV acquisition
Source: J Int AIDS Soc. 2023 Jul 13;26(Suppl 2):e26107. doi: 10.1002/jia2.26107 (PMC10805120; doi:10.1002/jia2.26107)
Supplement: Supplementary file 2 — Supporting Information Appendix B: Results from rapid extraction of 38 articles briefly mentioning preferences for injectables compared with other PrEP modalities [file JIA2-26-e26107-s001.docx]

| ***Appendix B****. Results from rapid extraction of 38 articles briefly mentioning preferences for injectables compared with other PrEP modalities. Injectable PrEP was preferred (+), less preferred (-), and equally preferred (=) in comparison to the other modality (modality in comparison).* | | | | | | | |
| --- | --- | --- | --- | --- | --- | --- | --- |
| **First Author (year)** | **Article Type** | **Region** | **Sex** | **Other Group Identifiers** | **Preference for injectable PrEP** | **Modality in comparison** | **RRef.** |
| Akolo, M. et al. (2018) | Abstract | Sub-Saharan Africa | Female | FSW | + | Oral Pill | [102] |
| Assoumou, S. et al. (2021) | Peer-reviewed: Observational Study | North America | Female, Male | PWID | + | Oral Pill | [103] |
| Belludi, A. et al. (2021) | Peer-reviewed: Observational Study | South Asia | Female, Male | PWID | - | Oral Pill | [104] |
|  |  |  |  | MSM | = | Daily Oral Pill |  |
| Biello, K. B et al. (2018) | Peer-reviewed: Observational Study | North America | Male | MSM | + | Daily Oral Pill | [105] |
| Callander, D. et al. (2019) | Peer-reviewed: Observational Study | Europe and Central Asia | Male | MSM & Transgender men | - | Daily Oral Pill | [106] |
|  |  |  |  |  | + | Episodic Oral Pill |  |
| Chen, W. et al. (2021) | Peer-reviewed: Observational Study | East Asia and Pacific | Male | MSM | - | Daily Oral Pill | [107] |
| Eisingerich, A. B. et al. (2012) | Peer-reviewed: Observational Study | Sub-Saharan Africa, South Asia, Latin America and the Caribbean, Europe and Central Asia | Female, Male | FSW, MSM, PWID, HIV-partner in sero-discordant couples, young women | + | Daily Oral Pill | [108] |
|  |  |  |  |  | + | Episodic Oral Pill |  |
| Galka, J. M. et al. (2020) | Peer-reviewed: Observational Study | East Asia and Pacific | Female | Transgender Women | - | Daily Oral Pill | [109] |
| Goedel, W. C. et al. (2018) | Peer-reviewed: Observational Study |  |  | MSM | + | Daily Oral Pill | [110] |
|  |  |  |  |  | + | Episodic Oral Pill |  |
|  |  |  |  |  | + | Microbocide Gels |  |
| Hall, E. W. et al. (2016) | Peer-reviewed: Observational Study |  |  | MSM | + | Daily Oral Pill | [111] |
|  |  |  |  |  | - | Episodic Oral Pill |  |
|  |  |  |  |  | - | Microbocide Gels |  |
| Hambrick, H. R. et al. (2018) | Peer-reviewed: Observational Study | Europe and Central Asia | Male | MSM | + | Daily Oral Pill | [112] |
|  |  |  |  |  | + | Episodic Oral Pill |  |
|  |  |  |  |  | + | Microbocide Gels (Rectal) |  |
| Harling, G. et al. (2019) | Peer-reviewed: Observational Study | Sub-Saharan Africa | Female | Female bar workers | + | Daily Oral Pill | [113] |
|  |  |  |  |  | + | Vaginal Gel |  |
|  |  |  |  |  | + | Vaginal Ring |  |
| Huang, W. et al. (2019) | Peer-reviewed: Observational Study | East Asia and Pacific | Male | MSM | - | Microbicides (gel) | [114] |
|  |  |  |  |  | + | Daily Oral Pill |  |
| Kennedy, A. J. et al. (2020) | Abstract | North America | Unspecified | Providers of outpatients using medication for opioid use disorder | + | Oral pill (not specified) | [115] |
| Levy, M. et al. (2021) | Peer-reviewed: Observational Study | North America | Male | MSM | + | Daily Oral Pill | [116] |
| Levy, M. et al. (2017) | Peer-reviewed: Observational Study | North America | Male | MSM | + | Daily Oral Pill | [117] |
| Liu, A. Y. et al. (2019) | Abstract | North America | Female, Male | MSM, Transgender Women | - | Episodic Oral Pill | [118] |
|  |  |  |  |  | + | Pericoital rectal formulation |  |
| Maria, D. M. S. et al. (2017) | Abstract | North America | Female, Male | Homeless young adults | + | Daily Oral Pill | [119] |
| Mayer, K. et al. (2016) | Abstract | North America | Male | MSM | + | Infusible antibodies | [120] |
|  |  |  |  |  | + | Pericoital rectal formulation |  |
|  |  |  |  |  | + | Rectal douche |  |
|  |  |  |  |  | + | Daily Oral Pill |  |
| Mgbako, O. et al. (2019) | Peer-reviewed: Observational Study | Europe and Central Asia | Male | MSM in transactional sex | + | Daily Oral Pill | [121] |
|  |  |  |  |  | + | Episodic Oral Pill |  |
|  |  |  |  |  | + | Microbicide gels (rectal and/or penile) |  |
| Minnis, A.M. et al. (2019) | Peer-reviewed: Observational Study | Sub-Saharan Africa | Female | TRIO clinical trial participants+ community (product) naive | + | Daily Oral Pill | [122] |
| Minnis, A.M. et al. (2021) | Peer-reviewed: RCT | Sub-Saharan Africa | Female | TRIO clinical trial participants | + | MPT Tablets | [123] |
|  |  |  |  |  | + | MPT Rings |  |
| Nematadzira, T. et al. (2021) | Abstract | Sub-Saharan Africa | Female, Male | Young people | = | Oral pill (not specified) | [124] |
| Pereira, C.C.A. et al. (2021) | Abstract | Latin America & the Caribbean | Female, Male | MSM, Transgender women, non-binary | + | Oral pill (not specified) | [125] |
| Reisner, S. L. et al. (2021) | Peer-reviewed: Observational Study | North America | Male | MSM, Transgender Men | + | Daily Oral Pill | [126] |
|  |  |  |  |  | + | Microbicide gels (rectal and/or penile) |  |
|  |  |  |  |  | + | Rectal douche |  |
|  |  |  |  |  | + | Infusable antibodies |  |
| Schneider, K. E. et al. (2021) | Peer-reviewed: Observational Study | North America | Female, Male | PWID | + | IV infusion | [127] |
|  |  |  |  |  | + | Implants |  |
| Siedner, M. J. et al. (2018) | Peer-reviewed: Observational Study | Sub-Saharan Africa |  |  | + | Daily Oral Pill | [128] |
| Timmins, L. et al. (2021) | Peer-reviewed: Observational Study | North America | Male | Black, gay and bisexual MSM |  |  | [129] |
|  |  |  |  | Current PrEP Users | + | Daily Oral Pill |  |
|  |  |  |  | Naïve Prep User | - | Daily Oral Pill |  |
| Torres, T. S. et al. (2018) | Abstract | Latin America | Male | MSM | + | Daily Oral Pill | [130] |
|  |  |  |  |  | + | Episodic Oral Pill |  |
| Torres, T. et al. (2018) | Peer-reviewed: Observational Study | Latin America & the Caribbean | Male | MSM | = | Daily Oral Pill | [131] |
|  |  |  |  |  | + | Episodic Oral Pill |  |
| Torres, T. et al. (2019) | Peer-reviewed: Observational Study | Latin America & the Caribbean | Male | MSM (Brazil & Peru) | + | Daily Oral Pill | [132] |
|  |  |  |  | MSM (Mexico) | - | Daily Oral Pill |  |
| Torres, T. et al. (2019) | Peer-reviewed: Observational Study | Latin America & the Caribbean | Male | MSM | - | Daily Oral Pill | [133] |
|  |  |  |  |  | + | Episodic Oral Pill |  |
| Tran Viet, Ha et al. (2019) | Peer-reviewed: Observational Study | East Asia and Pacific | Male | MSM | - | Daily Oral Pill | [134] |
| Vickerman, P. et al. (2020) | Peer-reviewed: Observational Study | Sub-Saharan Africa | Female | Adult and adolescent women | + | Daily Oral Pill | [135] |
| Wahome, E. et al. (2019) | Abstract | Sub-Saharan Africa | Male | MSM | + | Daily Oral Pill | [136] |
| Wheelock, A. et al. (2013) | Peer-reviewed: Observational Study | East Asia and Pacific | Male | MSM | - | Daily Oral Pill | [137] |
| World Health Organization (2022) | Report | Sub-Saharan Africa, East Asia & Pacific, Europe and Central Asia, Latin America & the Caribbean, North American | Female, Male | Sex workers | participants stated that a range of PrEP dosing regimens and modalities should be available to sex workers due to individual values and preference | Daily Oral Pill | [138] |
| FSW: female sex workers; HIV: human immunodeficiency virus; MSM: men who have sex with men; PrEP: pre-exposure prophylaxis; PWID: persons who inject drugs | | | | | | | |

**Reference**

102. Akolo M, Kimani J, Gelman L. PrEP Implementation: Retention Challenges Among Female Sex Workers in Nairobi, Kenya. HIV Research for Prevention; October 21 - 25, 2018; Madrid, Spain2018.

103. Assoumou SA, Paniagua SM, Gonzalez P, Wang J, Beckwith CG, White LF, et al. HIV Pre-exposure Prophylaxis and Buprenorphine at a Drug Detoxification Center During the Opioid Epidemic: Opportunities and Challenges. AIDS Behav. 2021;25(8):2591-8.

104. Belludi A, McFall AM, Solomon SS, Celentano DD, Mehta SH, Srikrishnan AK, et al. Awareness of and willingness to use pre-exposure prophylaxis (PrEP) among people who inject drugs and men who have sex with men in India: Results from a multi-city cross-sectional survey. PLoS One. 2021;16(2):e0247352.

105. Biello KB, Mimiaga MJ, Santostefano CM, Novak DS, Mayer KH. MSM at Highest Risk for HIV Acquisition Express Greatest Interest and Preference for Injectable Antiretroviral PrEP Compared to Daily, Oral Medication. AIDS Behav. 2018;22(4):1158-64.

106. Callander D, Park SH, Al-Ajlouni YA, Schneider JA, Khan MR, Safren SA, et al. Condomless Group Sex Is Associated With HIV Pre-Exposure Prophylaxis Knowledge and Interest Uptake: A Cross-Sectional Study of Gay and Bisexual Men in Paris, France. AIDS Educ Prev. 2019;31(2):127-35.

107. Chen W, Ding Y, Chen J, Zhao P, Wang Z, Meng X, et al. Awareness of and Preferences for Preexposure Prophylaxis (PrEP) among MSM at High Risk of HIV Infection in Southern China: Findings from the T2T Study. Biomed Res Int. 2021;2021:6682932.

108. Eisingerich AB, Wheelock A, Gomez GB, Garnett GP, Dybul MR, Piot PK. Attitudes and acceptance of oral and parenteral HIV preexposure prophylaxis among potential user groups: a multinational study. PLoS One. 2012;7(1):e28238.

109. Galka JM, Wang M, Azwa I, Gibson B, Lim SH, Shrestha R, et al. Willingness to Use Pre-Exposure Prophylaxis (PrEP) for HIV Prevention and PrEP Implementation Preferences Among Transgender Women in Malaysia. Transgend Health. 2020;5(4):258-66.

110. Goedel WC, Schneider JA, Hambrick HR, Kreski NT, Morganstein JG, Park SH, et al. Are Anal Sex Roles Associated with Preferences for Pre-Exposure Prophylaxis Administration Modalities Among Men Who Have Sex with Men? Arch Sex Behav. 2018;47(7):2123-33.

111. Hall EW, Heneine W, Sanchez T, Sineath RC, Sullivan P. Preexposure Prophylaxis Modality Preferences Among Men Who Have Sex With Men and Use Social Media in the United States. J Med Internet Res. 2016;18(5):e111.

112. Hambrick HR, Park SH, Schneider JA, Mayer KH, Carrico AW, Sherman SE, et al. Poppers and PrEP: Use of Pre-exposure Prophylaxis Among Men Who Have Sex with Men Who Use Inhaled Nitrites. AIDS Behav. 2018;22(11):3658-72.

113. Harling G, Muya A, Ortblad KF, Mashasi I, Dambach P, Ulenga N, et al. HIV risk and pre-exposure prophylaxis interest among female bar workers in Dar es Salaam: cross-sectional survey. BMJ Open. 2019;9(e023272).

114. Huang W, Wu D, Ong JJ, Smith MK, Pan S, Yang F, et al. Prepared for PrEP: preferences for HIV pre-exposure prophylaxis among Chinese men who have sex with men in an online national survey. BMC Infect Dis. 2019;19(1):1057.

115. Kennedy AJ, Hassan IF, de Abril Cameron F, Gobao V, Edelman J, Ho K, et al. Providing HIV pre-exposure prophylaxis to patients within an opiod treatment program: A qualitative study. Annual Meeting of the Society of General Internal Medicine; Jul2020. p. 1-779.

116. Levy ME, Agopian A, Magnus M, Rawls A, Opoku J, Kharfen M, et al. Is Long-Acting Injectable Cabotegravir Likely to Expand PrEP Coverage Among MSM in the District of Columbia? J Acquir Immune Defic Syndr. 2021;86(3):e80-e2.

117. Levy ME, Patrick R, Gamble J, Rawls A, Opoku J, Magnus M, et al. Willingness of community-recruited men who have sex with men in Washington, DC to use long-acting injectable HIV pre-exposure prophylaxis. PLoS One. 2017;12(8):e0183521.

118. Liu A, Coleman K, Walker N, Vittinghoff E, Turner C, Vinson J, et al. Assessing the prep continuum in the san francisco bay area: The quickie mobile survey. Conference on Retroviruses and Opportunistic Infections; March 4-7, 2019; Seattle, WA2019.

119. Santa Maria DM, Narendorf SC, Barman-Adhikari A, Petering R, Flash CA. Implications for Prep Uptake in Homeless Young Adults: A Mixed-Methods Study. Journal of Adolescent Health. 2017;60(2):S25-S6.

120. Mayer K, Biello K, Coffey-Esquivel J, Novak D, Mimiaga M. PrEP Knowledge, Uptake, and Comparative Interest in New Prevention Modalities in an On-line Sample of American Men who Have Sex with Men (MSM). HIV Research for Prevention; October 17-21, 2016; Chicago, IL, USA2016.

121. Mgbako O, Park SH, Mayer KH, Schneider JA, Goedel WC, Hambrick HR, et al. Transactional Sex and Preferences for Pre-Exposure Prophylaxis (PrEP) Administration Modalities Among Men Who Have Sex With Men (MSM). J Sex Res. 2019;56(4-5):650-8.

122. Minnis AM, Browne EN, Boeri M, Agot K, Van der Straten A, Ahmed K, et al. Young Women’s Stated Preferences for Biomedical HIV Prevention: Results of a Discrete Choice Experiment in Kenya and South Africa. J Acquir Immune Defic Syndr. 2019;80(4).

123. Minnis AM, Krogstad E, Shapley-Quinn MK, Agot K, Ahmed K, Danielle Wagner L, et al. Giving voice to the end-user: input on multipurpose prevention technologies from the perspectives of young women in Kenya and South Africa. Sex Reprod Health Matters. 2021;29(1):1927477.

124. Nematadzira T, Weiss H, Stranix-Chibanda L, Bere T, Ssemata A, Muhumuza R, et al. Attitudes towards PrEP and correlates of common mental disorder symptoms and substance use among young people. HIV Research for Prevention; January 27-28, 2021; February 3-4, 2021; Virtual2021.

125. Pereira CCA, Torres TS, Luz PM, Hoagland B, Farias A, Brito JDU, et al. Preferences for pre-exposure prophylaxis (PrEP) among sexual and gender minorities: a discrete choice experiment in Brazil. Lancet Reg Health Am. 2023;19:100432.

126. Reisner SL, Moore CS, Asquith A, Pardee DJ, Mayer KH. The Pre-Exposure Prophylaxis Cascade in At-Risk Transgender Men Who Have Sex with Men in the United States. LGBT Health. 2021;8(2):116-24.

127. Schneider KE, White RH, O'Rourke A, Kilkenny ME, Perdue M, Sherman SG, et al. Awareness of and interest in oral pre-exposure prophylaxis (PrEP) for HIV prevention and interest in hypothetical forms of PrEP among people who inject drugs in rural West Virginia. AIDS Care. 2021;33(6):721-8.

128. Siedner MJ, Hettema A, Hughey A, Oldenburg CE, Kohler S, Barnighausen K, et al. Preference for injectable over oral HIV pre-exposure prophylaxis in public-sector primary-care clinics in Swaziland. AIDS. 2018;32(11):1541-2.

129. Timmins L, Schneider JA, Chen YT, Goedel WC, Brewer R, Callander D, et al. Sexual Identity, Sexual Behavior and Pre-exposure Prophylaxis in Black Cisgender Sexual Minority Men: The N2 Cohort Study in Chicago. AIDS Behav. 2021;25(10):3327-36.

130. Torres TS, De Boni RB, de Vasconcelos M, Luz PM, Hoagland B, Moreira RI, et al. Comparing the characteristics of Brazilian MSM using app for sexual encounters. Conference on Retroviruses and Opportunistic Infections; March 4-7, 2018; Boston, MA, USA2018.

131. Torres TS, De Boni RB, de Vasconcellos MT, Luz PM, Hoagland B, Moreira RI, et al. Awareness of Prevention Strategies and Willingness to Use Preexposure Prophylaxis in Brazilian Men Who Have Sex With Men Using Apps for Sexual Encounters: Online Cross-Sectional Study. JMIR Public Health Surveill. 2018;4(1):e11.

132. Torres TS, Konda KA, Vega-Ramirez EH, Elorreaga OA, Diaz-Sosa D, Hoagland B, et al. Factors Associated With Willingness to Use Pre-Exposure Prophylaxis in Brazil, Mexico, and Peru: Web-Based Survey Among Men Who Have Sex With Men. JMIR Public Health Surveill. 2019;5(2):e13771.

133. Torres TS, Luz PM, De Boni RB, de Vasconcellos MTL, Hoagland B, Garner A, et al. Factors associated with PrEP awareness according to age and willingness to use HIV prevention technologies: the 2017 online survey among MSM in Brazil(). AIDS Care. 2019;31(10):1193-202.

134. Ha TV, Chockalingam L, Giang LM, Krishnan A, Bhadra M, Miller WC, et al. Awareness of HIV pre-exposure prophylaxis (PrEP) among men who have sex with men in Hanoi, Vietnam. Southeast Asian Journal for Tropical Medicine in Public Health. 2019;50(3).

135. Vickerman P, Quaife M, Kilbourne-Brook M, Mvundura M, Eakle R, Terris-Prestholt F. HIV prevention is not all about HIV - using a discrete choice experiment among women to model how the uptake and effectiveness of HIV prevention products may also rely on pregnancy and STI protection. BMC Infect Dis. 2020;20(1):704.

136. Wahome E, Mwashigadi G, Kombo B, Kimani M, van de Elst EM, Mohamed K, et al. Factors associated with refusing or stopping prep among at-risk MSM in Kenya. Conference on Retroviruses and Opportunistic Infections; March 4-7, 2019; Seattle, WA, USA2019.

137. Wheelock A, Eisingerich AB, Ananworanich J, Gomez GB, Hallett TB, Dybul MR, et al. Are Thai MSM willing to take PrEP for HIV prevention? An analysis of attitudes, preferences and acceptance. PLoS One. 2013;8(1):e54288.

138. Organization WH. Web Annex B. Values and preferences report. In: Consolidated guidelines on HIV, viral hepatitis and STI prevention, diagnosis, treatment and care for key populations. Geneva; 2022. Report No.: Licence: CC BY-NC-SA 3.0 IGO.
